# Multifunctional and endogenous stimuli-responsive vinblastine sulfate/manganese dioxide nanodrugs for enhancing chemotherapeutic efficacy against hypoxic tumors

**DOI:** 10.1016/j.mtbio.2025.102229

**Published:** 2025-08-23

**Authors:** Yong Geun Lim, Yeji Chang, Seon-Ju Park, Kyoung-Dong Kim, Kyeongsoon Park

**Affiliations:** Department of Systems Biotechnology, Chung-Ang University, Anseong, Gyeonggi, 17546, South Korea

**Keywords:** Vinblastine sulfate (VBL), Manganese dioxide (MnO_2_), Oxygen-generating materials, Tumor microenvironment (TME), Tumor hypoxia relief, Chemotherapy

## Abstract

Several solid tumors contain large hypoxic regions, diminishing drug responses and resulting in limited therapeutic outcomes. Vinblastine sulfate (VBL) targets the tubulin molecule, causing microtubule depolymerization and inducing mitotic arrest, leading to tumor regression. However, hypoxia causes microtubule depolymerization in tumor cells, reducing the number of intact microtubules for drug binding and diminishing the efficacy of microtubule-targeting agents like VBL. Therefore, to relieve tumor hypoxia and enhance chemotherapeutic responses to hypoxic tumors, we developed multifunctional and endogenous stimuli-responsive VBL/manganese dioxide (VBL/MnO_2_) nanodrugs. The VBL/MnO_2_ nanodrugs demonstrated colloidal stability and were readily degraded by endogenous stimuli, including acidic pH, hydrogen peroxide, and glutathione, resulting in the release of VBL and *in situ* oxygen generation. In addition, oxygen-generating VBL/MnO_2_ nanodrugs can alleviate hypoxia in tumor cells with high H_2_O_2_ levels after their intracellular uptake, thereby improving therapeutic efficacy better than VBL owing to enhanced drug responses and microtubule depolymerization. Furthermore, the accumulation of VBL/MnO_2_ nanodrugs in tumor tissues via passive targeting resulted in the effective reduction of hypoxia regions and hypoxia-inducible factor-1α expression *in vivo*. Owing to their remarkable ability to relieve tumor hypoxia, biocompatible VBL/MnO_2_ nanodrugs improve drug responses and tubulin aggregation in tumor tissues, ultimately enhancing tumor apoptosis and regression. These findings suggest that the VBL/MnO_2_ nanodrugs are a promising therapeutic strategy for enhancing chemotherapeutic responses to hypoxic tumors by ameliorating tumor hypoxia.

## Graphical abstract

**Figure:**
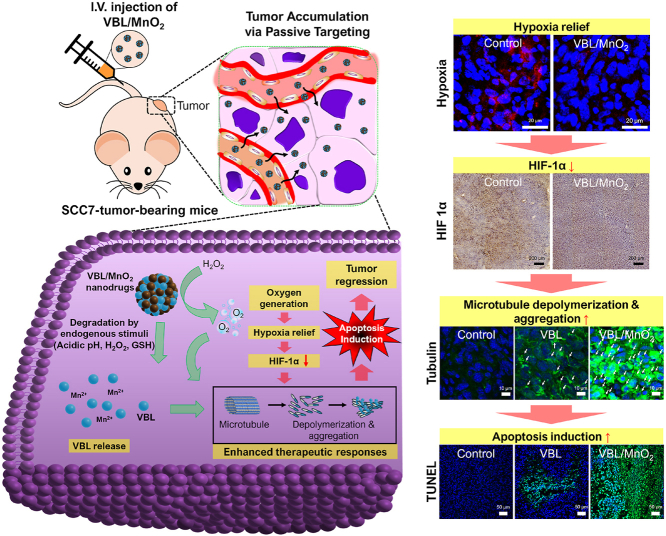

## 1 Introduction

Tumor microenvironment (TME) is an acidic milieu of a heterogeneous population of cells (e.g., tumor cells, various immune cell subtypes, and tumor-associated fibroblasts) and extracellular matrix constituents. It is also characterized by elevated levels of H_2_O_2_, glutathione (GSH), and hypoxia (low O_2_ content), attributed to aberrant growth and cellular metabolism within the tumor [[1], [2], [3]]. Hypoxia, in particular, in the TMEs promotes tumor progression, invasion, and metastasis, and high hypoxic regions inhibit effective drug responses to tumors, resulting in drug resistance to chemotherapy and ultimately leading to limited therapeutic responses [[4], [5], [6], [7]].

Vinblastine sulfate (VBL), an antitumor and microtubule-destabilizing drug, binds to the vinca site of tubulin, inhibiting microtubule formation, inducing mitotic arrest, and ultimately resulting in apoptotic cell death and tumor regression [8,9]. VBL has been utilized to treat Hodgkin and non-Hodgkin lymphomas as well as testicular, ovarian, breast, and head and neck cancers [8,10]. As a microtubule destabilizer, VBL induces microtubule depolymerization to form tubulin aggregation in normoxic tumor cells [11,12]. However, tumor hypoxia can interfere with the formation of tubulin aggregation, reducing drug response and causing ineffective treatment outcomes [13]. Furthermore, VBL causes severe side effects such as myelosuppression, mucositis, fever, anemia, and alopecia [8,14].

To reduce VBL's toxicity, various biocompatible nanocarriers have been developed for its delivery utilizing synthetic or natural polymers such as poly(lactide-*co*-glycolide) [15], poly(caprolactone)-grafted dextran copolymer [16], liposome [17], cyclodextrin [18], glycol chitosan-carboxymethyl β-cyclodextrins [19], and molecularly imprinted polymers [10,20]. Nano-scaled VBL delivery systems that employ synthetic or natural polymeric materials can achieve sustained drug release, improve bioavailability, and enhance tumor-targeting ability [10,17,18,21]. However, these VBL delivery systems lack hypoxia-alleviating materials and thus cannot address the issues of drug resistance and limited therapeutic responses to chemotherapy. Therefore, the development of smart and multifunctional materials that can deliver drugs within the TME and alleviate hypoxia to reduce drug resistance and enhance therapeutic responses to chemotherapy remains a significant challenge.

Oxygen-generating materials such as liquid peroxides, solid peroxides, and manganese dioxide (MnO_2_) can alleviate hypoxia in solid tumors or regenerative processes [[22], [23], [24]]. Among them, MnO_2_-based nanomaterials are readily degraded by endogenous stimuli (e.g., acidic pH, high H_2_O_2_, and high GSH) that can generate oxygen *in situ* and alleviate hypoxia in tumors [[25], [26], [27], [28]]. Furthermore, their hydrophobic surface property [29], makes MnO_2_ materials viable delivery carriers of hydrophobic drugs (e.g., taxol, chlorin e6 (Ce6), doxorubicin, and curcumin) [[30], [31], [32]]. MnO_2_-based nanomaterials degrade into water-soluble Mn^2+^ ions that are rapidly eliminated via renal excretion, thereby minimizing long-term systemic toxicity concerns [33,34]. Hence, endogenous stimuli-responsive MnO_2_ nanomaterials have been developed as smart drug carriers to target the TME, generate oxygen in hypoxic tumors, and enhance chemotherapy and photodynamic therapy responses [22,23,35].

Although MnO_2_ nanomaterials have promising multifunctional properties, many reported MnO_2_ nanostructures, including nanoparticles, nanosheets, and nanocomposites, struggle with limited drug loading capacity and controlled drug release [34,36,37]. To overcome these challenges, hollow nanostructures such as hollow mesoporous silica and hollow MnO_2_, characterized by mesoporous shells and large internal cavities, have been developed as platforms that can load high quantities of therapeutic agents [34,38]. However, the complex and multi-step synthetic procedures needed for these hollow architectures inhibit scalable production and clinical translation. Therefore, there is a need for novel, facile fabrication methods that enable the production of nanomedicines combining high drug-loading capacity with simplicity of synthesis.

**Scheme 1:**
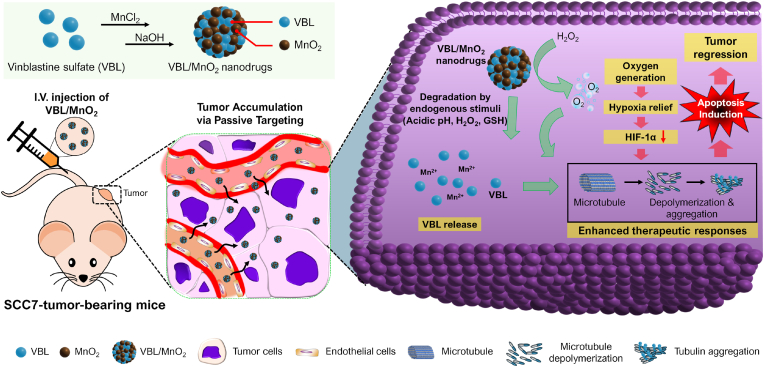
Schematic of multifunctional and endogenous stimuli-responsive VBL/MnO_2_ nanodrugs for enhancing chemotherapeutic effects in hypoxic tumors. The VBL/MnO_2_ nanodrugs were prepared via a simple reaction of VBL and MnCl_2_ under alkaline pH conditions. The systemically administered VBL/MnO_2_ nanodrugs were accumulated in tumors via passive targeting. The internalized VBL/MnO_2_ nanodrugs in tumor cells were readily degraded by endogenous stimuli, such as acidic pH, H_2_O_2_, or GSH, followed by the subsequent release of VBL and oxygen generation to relieve hypoxia. In addition, the released VBL and oxygen generation in tumor cells relieve tumor hypoxia and reduce HIF-1α levels, improving drug responses and tubulin aggregation, ultimately resulting in the effective induction of apoptosis and tumor regression.

To address the limitations of conventional VBL chemotherapy and simplify the fabrication of MnO_2_ nanodrugs with high drug-loading capacity, we developed a novel strategy to synthesize MnO_2_-based VBL (VBL/MnO_2_) nanodrugs via direct intermolecular coordination between Mn^2+^ ions and the metal-binding moieties of VBL. Unlike previously reported MnO_2_-based nanomaterials that often require complex synthesis and achieve lower drug loading efficiencies, our method involves a simple reaction of VBL and MnCl_2_ under alkaline conditions. The resulting VBL/MnO_2_ nanodrugs are multifunctional and responsive to endogenous tumor stimuli. They are designed to reduce systemic toxicity, target the hypoxic TME, enable smart and controlled VBL release, and alleviate tumor hypoxia. Together, these features enhance drug response and improve therapeutic outcomes against hypoxic tumors. Following systemic administration, the nanodrugs accumulated in tumors via passive targeting. The internalized nanodrugs in tumor cells subsequently released VBL and Mn^2+^ following biodegradation by endogenous stimuli, such as acidic pH, H_2_O_2_, or GSH, thereby producing oxygen to relieve hypoxia. Finally, we evaluated *in vitro* and *in vivo* therapeutic outcomes focusing on oxygenation of hypoxic tumors, inhibition of hypoxia-inducible factor (HIF)-1α (a key mediator of hypoxia signaling) expression, and enhancement of drug responses and tubulin aggregation in tumors. These resulted in the effective induction of apoptotic cell death and tumor regression (Scheme 1).

## 2 Materials and methods

### 2.1 Materials

VBL was purchased from Biosynth Ltd. (Berkshire, UK). Manganese (Ⅱ) chloride tetrahydrate (MnCl_2_·4H_2_O), diethylenetriamine pentaacetic acid (DTPA), potassium permanganate (KMnO_4_), sodium hydroxide (NaOH), potassium bromide (KBr), hydrogen peroxide solution (H_2_O_2_, 30 wt%), Tween 20, L-GSH, dimethyl sulfoxide (DMSO), and 3.7 % formaldehyde solution were procured from Sigma-Aldrich (St. Louis, MO, USA). Phosphate-buffered saline (PBS) and 0.9 % normal saline (NS) solution were purchased from Lonza (Walkersville, MD, USA) and Dai Han Pharm (Seoul, Korea), respectively. Dulbecco's Modified Eagle Medium (DMEM) without phenol red, RPMI 1640, fetal bovine serum (FBS), and Dulbecco's Phosphate-Buffered Saline (DPBS) were obtained from Welgene Inc. (Seoul, Korea). Ce6 was purchased from Frontier Scientific (Logan, UT, USA). Cell-counting kit-8 (CCK-8) was obtained from Dojindo Molecular Technologies, Inc. (Kumamoto, Japan). In addition, 4,6-diamidino-2-phenylindole (DAPI) was obtained from GBI Labs (Bothell, WA, USA). Image-iT™ Green Hypoxia Reagent, Image-iT™ Red Hypoxia Reagent, and GAPDH antibody were obtained from Thermo Fisher Scientific Inc. (MA, USA). Alexa Fluor® 488 Anti-alpha Tubulin antibody was obtained from Abcam (UK). Anti-HIF-1α and cleaved caspase-3 antibody were purchased from Novus Biologicals (USA). Anti-rabbit IgG secondary antibody was procured from Bio-Rad (CA, USA). RIPA lysis buffer, universal protease inhibitor cocktail, and Westglow™ ECL Chemiluminesent substrate were obtained from Biomax (Guri, Korea).

### 2.2 Synthesis and characterizations of VBL/MnO_2_ nanodrugs

VBL (15 mg, 16.5 μmol) and MnCl_2_·4H_2_O (13.06 mg, 66 μmol) were reacted in 10 mL of deionized water (DW) for 10 min, and then 1 M NaOH (400 μL) was added to the reaction solution to adjust the pH to 11. After a 20-min reaction, the prepared VBL/MnO_2_ nanodrugs were collected via centrifugation (3500 rpm, 10 min) and washed five times with DW to discard the unreacted VBL and NaOH. Subsequently, the purified VBL/MnO_2_ nanodrugs were redispersed in 10 mL of DW and stored at 4 °C for further experiments.

Particle size analyses, including size distribution, hydrodynamic average size, Z-average size, and polydispersity index (PDI) value of the purified VBL/MnO_2_ nanodrugs, were conducted utilizing a particle size analyzer (SZ-100, Horiba, Kyoto, Japan). A 10-fold diluted VBL/MnO_2_ nanodrug solution was prepared and then dispersed with an Ultrasonic Processor (KFS-150N, Korea Process Technology Co., Ltd., Seoul, Korea) for 5 min at 22.5 W. Furthermore, a 10-fold diluted VBL/MnO_2_ nanodrug solution was dropped onto a carbon-coated copper grid (Carbon Type-B, Ted Pella Inc., Redding, CA, USA) and air-dried in a fume hood for TEM analysis. The morphology of the dried VBL/MnO_2_ nanodrug was then examined with a field-emission transmission electron microscope (FE-TEM, JEM-F200, Tokyo, Japan). In addition, the zeta potential of VBL and VBL/MnO_2_ nanodrugs was measured with a Zetasizer (Malvern Panalytical Ltd., Malvern, UK).

The absorption spectrum of the VBL/MnO_2_ nanodrug was recorded with a UV/Vis spectrophotometer (NEO-S490, NEOGEN, Inc., Daejeon, Korea). The amount of VBL in the VBL/MnO_2_ nanodrug was further calculated from the standard curve obtained for VBL (Y = 16.13x + 0.0077; R^2^ = 0.9995) with the same instrument. The Mn^2+^ content in the VBL/MnO_2_ nanodrug was determined utilizing an inductively coupled plasma optical emission spectrometer (ICP-OES, Optima 7300 DV, PerkinElmer, USA) after the treatment with nitric acid and H_2_O_2_. The VBL loading content within the VBL/MnO_2_ nanodrugs was then calculated with the following equation:

$$\text{VBL}\;l\text{oading}\;\text{content}\;\left(\text{wt}\%\right)=\frac{\text{Weight}\;\text{of}\;\text{loaded}\;\text{VBL}}{{\text{Weight}\;\text{of}\;\text{the}\;\text{VBL}/\text{MnO}}_{2}\;\text{nanodrugs}\;}\times 100\%$$

To confirm the synthesis of the VBL/MnO_2_ nanodrug, the lyophilized VBL/MnO_2_ nanodrug was analyzed via Fourier transform-infrared spectroscopy (FT-IR, Shimadzu 8400S, Kyoto, Japan). Spectra were recorded at a resolution of 4 cm^−1^, between 4000 and 400 cm^−1^, employing the KBr pellet method. In addition, X-ray photoelectron spectroscopy (XPS) analysis was conducted with a K-alpha+ (Thermo Scientific, MA, USA) equipped with a Cu-Kα radiation source.

To assess the stability of the nanodrug in various aqueous conditions, the 10-fold diluted VBL/MnO_2_ nanodrugs from the stock solution were prepared in four different solutions: DW, DMEM containing 10 % FBS (10 % FBS), 0.9 % NS, and PBS 1X (pH 7.4). The dispersed VBL/MnO_2_ nanodrugs in each solution were stored at 25 °C, and their Z-average sizes and photos were acquired at scheduled time intervals (0, 1, 3, 6, 12, 24, 48, 72, and 96 h). In addition, for long-term stability testing, 10-fold diluted VBL/MnO_2_ nanodrugs diluted in DW were stored at 4 °C, and their Z-average sizes were monitored over 30 days at the scheduled time intervals (0, 3, 5, 7, 9, 11, 15, 30 days).

### 2.3 Degradation and oxygen generation of VBL/MnO_2_ nanodrugs under TME-mimic conditions

To investigate the degradation behavior of the VBL/MnO_2_ nanodrugs (10-fold dilution from the stock solution) under TME-mimic conditions, reactions with the VBL/MnO_2_ nanodrugs were observed in different physiological microenvironment conditions (i.e., pH, H_2_O_2_, and GSH). The detailed conditions of each group were as follows: PBS 7.4, PBS 7.4 + 200 μM H_2_O_2_, PBS 7.4 + 2 mM GSH, PBS 6.0, PBS 6.0 + 200 μM H_2_O_2_, and PBS 6.0 + 2 mM GSH. Changes in absorbance were monitored at 320 nm for each group utilizing a UV/Vis spectrophotometer for 10 min.

To assess the oxygen generation from VBL/MnO_2_ nanodrugs in hypoxic conditions, 10 mL of the VBL/MnO_2_ nanodrug solution and 10 mL of H_2_O_2_ solutions at different concentrations (PBS 7.4 with 0, 200, 400, and 800 μM of H_2_O_2_) were purged with N_2_ for 10 min. The VBL/MnO_2_ nanodrug solution was then added to each H_2_O_2_ solution with different concentrations (final H_2_O_2_ concentration of the mixture: 0, 100, 200, and 400 μM) and reacted for 10 min. During the 10-min reaction, dissolved oxygen levels were measured with an Oakton® DO 6+ Dissolved Oxygen Meter (EW-35643-15, Cole-Parmer®, Vernon Hills, IL, USA).

### 2.4 *In vitro* intracellular H_2_O_2_ levels of SCC7 cells under normoxic or hypoxic conditions

Squamous cell carcinoma of head and neck (SCC7) was obtained from the American Type Culture Collection (ATCC, Rockville, MD, USA). SCC7 cells were cultured in an RPMI 1640 medium containing 10 % FBS and 1 % penicillin-streptomycin at 37 °C and 5 % CO_2_ in a humid environment.

To evaluate the intracellular H_2_O_2_ levels of SCC7 cells cultured under normoxic or hypoxic conditions, cells were seeded at a density of 3 × 10^4^ cells/well in 4-well culture plates and incubated for 24 h. After washing, the cells were further incubated for 24 h under a humidified 5 % CO_2_ incubator (Normoxia) or a N_2_-gas-purged, laboratory-made hypoxic chamber (Hypoxia), as previously reported [27]. Subsequently, the cultured cells were treated under two different conditions with 2′,7′-dichlorofluorescein diacetate (DCFDA, 10 μM) for 30 min. The cells were then fixed with a 3.7 % formaldehyde solution for 30 min, followed by cell nucleus staining utilizing the fluoromount solution containing 4′,6′-diamidino-2-phenylindole hydrochloride (DAPI) (SouthernBiotech, AL, USA). Intracellular H_2_O_2_ levels in SCC7 cells were detected with a customized confocal laser scanning microscope (CLSM), and their fluorescence intensities (FI) under two different conditions were quantified utilizing the ImageJ software (Ver 1.53a, NIH, MD, USA).

### 2.5 *In vitro* intracellular uptake of VBL/MnO_2_ nanodrugs under normoxic or hypoxic conditions

To confirm the intracellular uptake of VBL/MnO_2_ nanodrugs, the hydrophobic Ce6, utilized as a fluorescent dye, was also adsorbed onto the surface of VBL/MnO_2_ nanodrugs. Briefly, the VBL/MnO_2_ nanodrugs dispersed in 10 mL of DW were reacted with 1 mL of Ce6 solution (2.5 mg/mL, solubilized in DMSO) for 24 h. The resulting solution was purified three times by centrifugation (3500 rpm, 10 min) utilizing DW. The prepared Ce6-adsorbed VBL/MnO_2_ (Ce6/VBL/MnO_2_) nanodrugs were further stored at 4 °C *in vitro* and *in vivo* experiments. The absorption spectra of Ce6/VBL/MnO_2_ nanodrugs (equivalent to 0.00783 mg/mL of Ce6) and free Ce6 (0.00783 mg/mL) were recorded with a UV/Vis spectrophotometer. The amount of Ce6 in Ce6/VBL/MnO_2_ nanodrugs was determined with a standard curve obtained for Ce6 (Y = 43.673x + 0.01; R^2^ = 0.9999) by UV/Vis spectrophotometry. Further, the fluorescence spectra of the Ce6/VBL/MnO_2_ nanodrugs (equivalent to 0.015 mg/mL of Ce6) in PBS (pH 7.4) with or without 200 μM H_2_O_2_ were recorded with a fluorescence spectrophotometer (FS-2, Scinco, Seoul, Republic of Korea).

To assess the *in vitro* intracellular uptake of VBL/MnO_2_ nanodrugs, SCC7 cells (1 × 10^4^ cells/well) were cultured in 4-well cell culture plates for 24 h. Following normoxic or hypoxic conditioning (as described in Section 2.4), the SCC7 cells were treated with fresh media or Ce6/VBL/MnO_2_ nanodrugs containing 5 μM Ce6. After a 4-h incubation, the cells in each group were fixed with a 3.7 % formaldehyde solution, stained with DAPI, and imaged via customized CLSM systems. Fluorescence signals from randomly selected CLSM images were quantified utilizing ImageJ software.

### 2.6 *In vitro* hypoxia alleviation of VBL/MnO_2_ nanodrugs

Before assessing the hypoxia-alleviating effects of VBL/MnO_2_ nanodrugs, DTPA/MnO_2_ nanoparticles (referred to as MnO_2_; Z-average size = 259.5 nm, Zeta potential = −18.5 mV) were prepared according to the established protocol [39]. These nanoparticles demonstrated excellent dispersibility and colloidal stability and were therefore adopted as the MnO_2_ treatment group.

To assess the *in vitro* hypoxia-alleviation ability of VBL/MnO_2_ nanodrugs, SCC7 cells (1 × 10^4^ cells/well) were prepared under normoxic or hypoxic conditions as described in Section 2.4. The as-prepared cells were then treated with free VBL (5 μM), MnO_2_ (6.1 μM), or VBL/MnO_2_ nanodrugs (equivalent to 5 μM VBL and 6.1 μM MnO_2_) for 4 h. After washing with DPBS, they were treated with Image-iT™ Green Hypoxia Reagent (5 μM) for 30 min per the manufacturer's instructions. After washing cells with DPBS and replacing the culture medium with a fresh one, the cells in the normoxia group were further incubated for 4 h in a humidified 5 % CO_2_ atmosphere. In contrast, the cells in the hypoxia group were incubated for 20 min in a N_2_-purged lab-made hypoxic chamber, followed by additional incubation for 4 h in a humidified 5 % CO_2_ atmosphere. After washing with DPBS, the cells were fixed with 3.7 % formaldehyde, stained with DAPI, and imaged with a CLSM. Average fluorescence intensity was quantified from randomly selected images utilizing the ImageJ software.

To further assess the hypoxia-alleviating effect of VBL/MnO_2_ nanodrugs in a 3D tumor spheroid model, SCC7 cells were seeded at a density of 1 × 10^3^ cells per well in ultra-low-attachment U-bottom 96-well plates (Costar®, USA) and incubated for 48 h to form uniform spheroids. To minimize accidental aspiration and spheroid loss during medium exchange, only half of the total medium volume was replaced each time. After washing with fresh medium, spheroids were incubated under hypoxic conditions as described in Section 2.4, and then treated with free VBL (5 μM), MnO_2_ (6.1 μM), or VBL/MnO_2_ nanodrugs (5 μM VBL and 6.1 μM MnO_2_) for 24 h. Following triple washing, spheroids were incubated with Image-iT™ Green Hypoxia Reagent (5 μM) for 1 h per the manufacturer's instructions. After additional washes, the medium was replaced with fresh medium, and spheroids were incubated for 20 min in an N_2_-purged lab-made hypoxic chamber, followed by a 4-h incubation in a humidified atmosphere containing 5 % CO_2_. Spheroids were then washed with DPBS, fixed in 3.7 % formaldehyde for 30 min, and transferred onto glass slides for DAPI counterstaining. Z-stack projection images comprising 40 optical sections, each 2.5 μm thick were acquired via CLSM imaging.

### 2.7 *In vitro* anticancer effects of VBL/MnO_2_ nanodrugs in cells

To investigate *in vitro* anticancer effects of VBL/MnO_2_ nanodrugs, SCC7 cells (5 × 10^3^ cells/well) were prepared in 96-well cell culture plates under normoxic or hypoxic conditions, as described in Section 2.4. The as-prepared SCC7 cells were treated with free VBL, MnO_2_, or VBL/MnO_2_ nanodrugs at various concentrations (0, 2, 5, 10, and 20 μM VBL; 0, 2.44, 6.1, 12.2, and 24.4 μM MnO_2_) for 24 h. After washing the cells with DPBS, they were exposed to a CCK-8 reagent for 4 h. The optical densities at 450 nm were then measured with a microplate reader (Multiskan Go, Thermo Fisher Scientific).

To validate the effects of VBL/MnO_2_ nanodrugs on cell cycle arrest, SCC7 cells (1 × 10^6^ cells/well) were seeded in 90-mm cell culture dishes and incubated at 37 °C in a humidified 5 % CO_2_ atmosphere for 24 h. The cells were then exposed to hypoxic conditions as described in Section 2.4, followed by treatment with free VBL (5 μM), MnO_2_ (6.1 μM), or VBL/MnO_2_ nanodrugs (equivalent to 5 μM VBL and 6.1 μM MnO_2_) for 24 h. After treatment, they were washed with DPBS, detached utilizing 1X trypsin-EDTA solution, and resulting cell suspensions were collected by centrifugation at 1000 rpm for 5 min at 4 °C. The supernatant was then discarded. The cell pellets were fixed in 1 mL of pre-chilled 70 % ethanol and stored at 4 °C for 24 h. Following careful washing with pre-cooled DPBS, the fixed cells were incubated with propidium iodide (PI) solution containing RNase A at 37 °C for 30 min. Cell cycle distribution was analyzed with a BD LSRFortessa™ X-20 flow cytometer (BD Biosciences, San Jose, CA), and data from approximately 10,000 gated cells were processed utilizing FACSDiva software (version 8.0.1).

### 2.8 *In vitro* microtubule depolymerization of VBL/MnO_2_ nanodrugs in normoxic or hypoxic cells

To assess the time-dependent effects of hypoxia on microtubule depolymerization in SCC7 cells, the cells (2 × 10^4^ cells/well) were seeded in 8-well culture plates for 24 h. The prepared cells were incubated under normoxic or various hypoxic conditions (1 h, 4 h, or 24 h) utilizing the CO_2_ incubator or hypoxic chamber, respectively. After washing the cells with DPBS, as-prepared SCC7 cells were fixed with a 3.7 % formaldehyde solution for 30 min. They were then washed with DPBS, followed by treatment with permeabilization buffer (0.2 % Triton X-100) for 10 min. They were also incubated with 100 μL of Alexa Fluor® 488 Anti-alpha Tubulin antibody (1:200 dilution) for 1 h. After staining their nuclei with DAPI, the fluorescence images were captured via a customized CLSM system to visualize hypoxia-induced microtubule depolymerization. Then, the FIs of each group were quantified utilizing an ImageJ software.

To evaluate the microtubule depolymerization induced by VBL/MnO_2_ nanodrugs, SCC7 cells (2 × 10^4^ cells/well) in 8-well culture plates were incubated under normoxic or hypoxic conditions for 1 h. The as-prepared SCC7 cells, either in normoxic or hypoxic conditions, were treated with free VBL (5 μM), MnO_2_ (6.1 μM), or VBL/MnO_2_ nanodrugs (equivalent to 5 μM VBL and 6.1 μM MnO_2_) in N_2_-purged medium for 4 h. After washing with DPBS, the cells were fixed with a 3.7 % formaldehyde solution for 30 min and then treated with permeabilization buffer (0.2 % Triton X-100) for 10 min. They were then exposed to 100 μL of Alexa Fluor® 488 Anti-alpha Tubulin antibody (1:200 dilution) for 1 h. Subsequently, the nuclei of the washed cells were counterstained with DAPI. The fluorescence images were acquired to visualize microtubule depolymerization and tubulin aggregation in the cells utilizing a customized CLSM system.

For detailed observation of microtubule morphology via stochastic optical reconstruction microscopy (STORM), SCC7 cells (2 × 10^4^ cells/well) were seeded in μ-slide 8-well high glass bottom chambers (IBIDI GMBH) and incubated under normoxic or hypoxic conditions for 1 h. The cells were treated with N_2_-purged medium containing free VBL (5 μM), MnO_2_ (6.1 μM), or VBL/MnO_2_ nanodrugs (equivalent to 5 μM VBL and 6.1 μM MnO_2_) for 4 h. After treatment, the cells were fixed, permeabilized, and stained with Alexa Fluor® 488 Anti-alpha Tubulin antibody (1:200 dilution) as described above. Following washing with DPBS, the cells were imaged in STORM buffer (ONI, UK) utilizing a Nanoimager system equipped with a 488-nm laser (ONI, UK). Imaging conditions included an illumination angle of 52°, an exposure time of 30 ms, and acquisition of 10,000 images per field of view. The acquired images were then processed utilizing CODI software (ONI, UK).

To validate VBL/MnO_2_ nanodrug-induced microtubule depolymerization in a 3D tumor spheroid model, SCC7 cells (1 × 10^3^ cells/well) were seeded in ultra-low-attachment U-bottom 96-well plates (Costar®) and incubated for 48 h to allow spheroid formation. After washing, spheroids were incubated under hypoxic conditions as described in Section 2.4. They were then treated with N_2_-purged medium containing free VBL (5 μM), MnO_2_ (6.1 μM), or VBL/MnO_2_ nanodrugs (equivalent to 5 μM VBL and 6.1 μM MnO_2_) for 24 h. Following treatment, spheroids were washed and fixed with 3.7 % formaldehyde for 1 h, permeabilized with 0.2 % Triton X-100 for 10 min, and stained with Alexa Fluor® 488 anti-α-tubulin antibody (1:200 dilution) for 1 h. After transferring the spheroids onto glass slides, they were counterstained with DAPI. Z-stack projection images (40 optical sections, each 2.5 μm thick) were acquired via a customized CLSM system.

### 2.9 *In vivo* tumor accumulation and hypoxia alleviation effect of VBL/MnO_2_ nanodrugs

*In vivo* animal experiments were approved by the Institutional Animal Care and Use Committee of Chung-Ang University (A2022059). The *in vivo* studies were performed in compliance with the National Research Council's Guide for the Care and Use of Laboratory Animals. SCC7 cells (1 × 10^6^ cells/mouse) were subcutaneously injected into the flank of C3H mice (8-week-old, male; Doo Yeol Biotech, Seoul, Korea) to establish a tumor model. All *in vivo* experiments began when the tumor volume reached approximately 100–150 mm^3^.

To evaluate tumor accumulation, SCC7 tumor-bearing mice were systemically treated with PBS (n = 3) or Ce6/VBL/MnO_2_ nanodrugs (2 mg/kg of Ce6, n = 3) via the lateral tail vein. The mice were sacrificed to excise tumor tissues and major organs (e.g., liver, lung, spleen, and kidney) 24 h after systemic administration, and *ex vivo* fluorescence images of the excised tumors and major organs were acquired with a small animal imaging system (InVivo Smart-LF, VIEWORKS, Anyang, Korea). Furthermore, frozen sections of tumor tissues were imaged utilizing a custom-built CLSM, and the FIs of each group were quantified with ImageJ software.

SCC7-tumor-bearing mice were intravenously administered PBS (n = 3) or VBL/MnO_2_ nanodrugs (2 mg/kg of VBL) (n = 3) to verify *in vivo* hypoxia alleviation. After 22 h, all mice in each group received an additional Image-iT™ Red Hypoxia Reagent (200 nmol/mouse) via the lateral tail vein [40]. After 2 h, tumor tissues were excised from the mice in each group, and *ex vivo* fluorescence images and intensities of the tumor tissues in each group were analyzed with a small animal imaging system (InVivo Smart-LF). Furthermore, the frozen sections of tumor tissues were imaged with a custom-built CLSM. To further assess HIF-1α expression, the frozen sections of tumor tissues in each group were treated with an anti-HIF-1α antibody (Dilution ratio = 1:100) and incubated at 4 °C overnight. HIF-1α-stained sections were then washed with Tris-buffered saline containing Tween 20 (TBST) and analyzed with the Polink-2 HRP Plus Mouse DAB Detection System (GBI Labs, Bothell, WA, USA). All tissue slides were dehydrated, cleared, mounted, and observed under an automated slide scanner (Axio Scan Z1, Zeiss, Oberkochen, Germany).

### 2.10 *In vivo* anticancer effect

SCC7 tumor-bearing mice were established in C3H mice, as described in Section 2.8. When tumor volume reached 100–150 mm^3^, the mice intravenously received PBS (n = 5), VBL (n = 5, 2 mg/kg; n = 5, 4 mg/kg), and VBL/MnO_2_ nanodrugs (n = 5, equivalent to 2 mg/kg of VBL; n = 5, equivalent to 4 mg/kg of VBL) once every 2 days (five times in total). Each group's tumor volumes were recorded daily and calculated with the following equation: (length × width^2^)/2. On Day 10 post-treatment, the excised tumor tissues were weighed and cryo-sectioned. Sliced tissues (n = 3 per group) were stained with hematoxylin and eosin (H&E). Then, the stained tissues were observed with an automated slide scanner (Axio Scan Z1).

To verify the *in vivo* formation of tubulin aggregation, frozen sections were fixed with 4 % formaldehyde, treated with hydrogen peroxidase, and washed with 1X TBST buffer. Sliced tissues (n = 3/group) were then incubated with Alexa Fluor® 488 Anti-alpha Tubulin antibody (1:200 dilution) at 4 °C overnight. Tissue sections were carefully washed with 1X TBST buffer, and their nuclei were counterstained with a DAPI-containing fluorescence mounting medium. *In vivo* tubulin aggregation was observed in each group utilizing a custom-built CLSM.

Paraffin-embedded tissue sections were stained with a DeadEnd™ Fluorometric TUNEL System (Promega, USA) according to the manufacturer's protocol to detect the apoptosis. After deparaffinization, ethanol dehydration, and rehydration, the tissue sections were fixed with 4 % formaldehyde, treated with proteinase K for 10 min, and then immersed in a peroxide blocking solution for another 10 min. The sections were then treated with an equilibration buffer for 10 min, followed by incubation with the TdT enzyme at 25 °C for 1 h. Next, the sections were treated with 2X saline-sodium citrate (SSC) buffer (Stop buffer). After a 15-min treatment, their nuclei were counterstained with DAPI. Finally, apoptotic cell death in sliced tissues was observed with a custom-built CLSM.

To assess apoptosis induction by VBL/MnO_2_ nanodrugs, approximately 0.5 g of isolated SCC7 tumor tissues per treatment group were mechanically homogenized in 1 mL of ice-cold RIPA lysis buffer (Biomax, Guri, Korea) supplemented with a universal protease inhibitor cocktail (Biomax, Guri, Korea) for 10 min. The homogenates were centrifuged at 15,000 rpm for 25 min at 4 °C, and the supernatants were collected and stored at −80 °C until further analysis. Protein concentrations were determined by BCS assay. Equal amounts of protein (25 μg per lane) were separated by 15 % SDS-PAGE and transferred to 0.2 μm nitrocellulose membranes (Amersham) utilizing glycine-based transfer buffer. After blocking, membranes were incubated overnight at 4 °C with primary antibody against cleaved caspase-3 (1:500; Novus Biologicals). Membranes were then washed and incubated with horseradish peroxidase (HRP)-conjugated anti-rabbit IgG secondary antibody (1:5000; Bio-Rad) for 1 h at room temperature. Immunoreactive bands were detected with Westglow™ ECL chemiluminescent substrate (Biomax, Guri, Korea), with GAPDH (1:2000; Thermo Fisher Scientific) as the loading control. Band intensities were quantified utilizing ImageJ software and normalized to GAPDH expression to determine relative levels of cleaved caspase-3.

### 2.11 *In vivo* biosafety assay

To evaluate the *in vivo* biosafety, as-prepared SCC7-tumor-bearing mice were intravenously injected with PBS (n = 5), VBL (n = 5; 2 and 4 mg/kg), and VBL/MnO_2_ nanodrugs (n = 5; equivalent to 2 and 4 mg/kg of VBL) once every 2 days (five times in total). Mice body weights in all groups were recorded daily. On Day 10 post-injection, blood samples were acquired from the inferior vena cava of the mice under anesthesia with isoflurane (1 %, w/v, JW-Pharma, Korea) in 2 L of oxygen. The mice were then sacrificed, and major organs (liver, lung, spleen, kidney, and heart) were harvested for histological analysis. The serum was obtained by centrifugation (1500 g, 4 °C, 15 min) of the acquired blood samples without anticoagulant. To investigate the serum biochemistry, liver function was examined by measuring aspartate aminotransferase (AST), alanine transaminase (ALT), and alkaline phosphatase (ALP) levels. Kidney function was assessed based on serum uric acid (SUA) and creatinine (CREA) levels. All parameters were measured with an automatic analyzer (DRI-CHEM NX500i, FUJIFILM, Japan). For histological analysis, the harvested major organs were cryosectioned into 10-μm slices, stained with H&E, and observed with an automated slide scanner (Axio Scan Z1).

### 2.12 Statistical analysis

Data are presented as the means ± standard deviations (SDs). A one-way analysis of variance (ANOVA) was performed between the two groups utilizing SigmaPlot (ver. 15; Chicago, IL, USA). Statistical significance was determined by *P* values of less than 0.05, 0.01, or 0.001.

## 3 Results and discussion

### 3.1 Synthesis and characterization of VBL/MnO_2_ nanodrugs

**Fig. 1:**
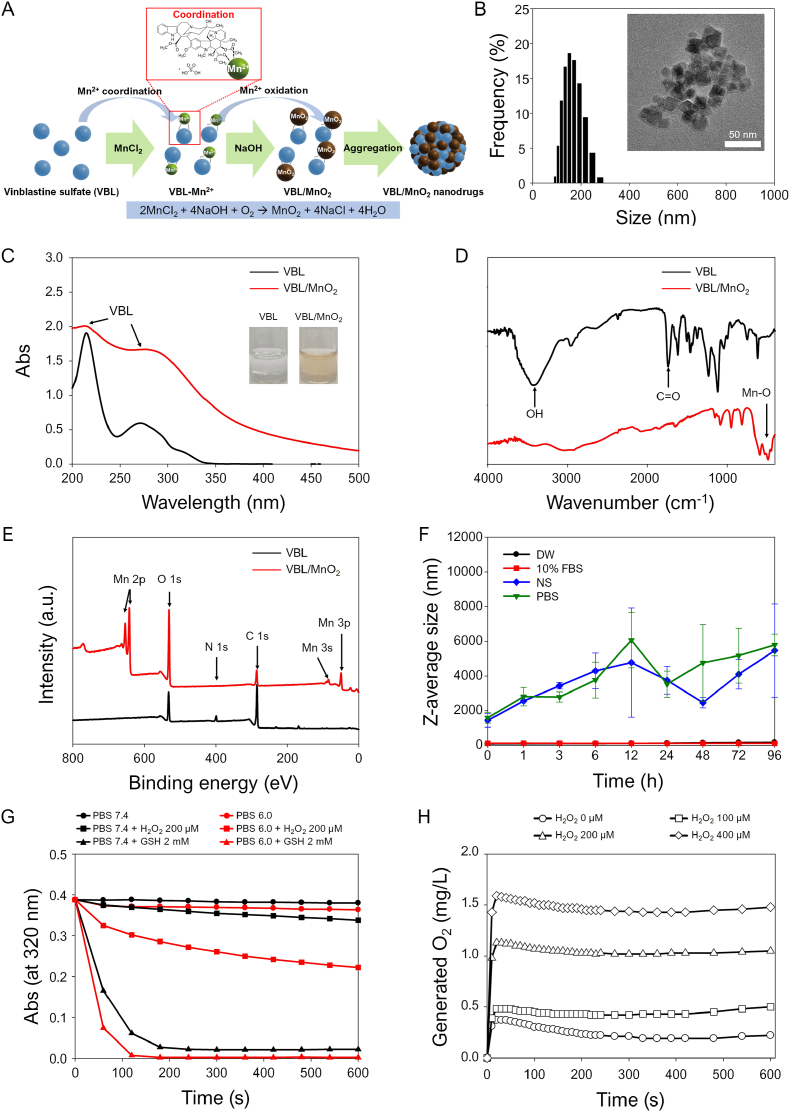
Synthesis and characterization of VBL/MnO_2_ nanodrugs. (A) Synthetic scheme of VBL/MnO_2_ nanodrugs. VBL/MnO_2_ nanodrugs were prepared by a simple reaction of vinblastine sulfate (VBL) with MnCl_2_ under alkaline pH. During the reaction under alkaline pH, Mn^2+^-coordinated VBL (VBL-Mn^2+^) was oxidized to produce VBL/MnO_2_ nanodrugs. (B) Size distribution of VBL/MnO_2_ nanodrugs determined by DLS. Inset: TEM image of VBL/MnO_2_ nanodrugs. Scale bar: 50 nm. (C) UV/Vis spectra of VBL and VBL/MnO_2_ nanodrugs. Inset: Photos of VBL and VBL/MnO_2_ nanodrug solution. (D) FT-IR spectra and (E) wide scan XPS spectra of VBL and VBL/MnO_2_ nanodrugs. (F) Z-average sizes of VBL/MnO_2_ nanodrugs stored in various solutions (i.e., Dulbecco's Modified Eagle's Medium (DMEM), 10 % fetal bovine serum (FBS), 0.9 % normal saline (NS), and phosphate-buffered saline (PBS) at pH 7.4), as measured at different time points. (G) *In vitro* degradation behaviors of VBL/MnO_2_ nanodrugs by endogenous stimuli of TME such as acidic pH, H_2_O_2_, and GSH, as determined by monitoring the absorbance at 320 nm for 10 min. (H) Oxygen generation from VBL/MnO_2_ nanodrugs during a 10-min reaction in N_2_-purged PBS (hypoxia) with different H_2_O_2_ concentrations (0−400 μM H_2_O_2_).

Fig. 1A represents the synthetic scheme of multifunctional and endogenous stimuli-responsive VBL/MnO_2_ nanodrugs under alkaline pH conditions. O_2_ of vindoline units in VBL could be coordinated with Mn^2+^ ions via ion-dipole interactions [41,42]. Hence, the Mn^2+^-coordinated VBL (VBL-Mn^2+^) would be formed by simply mixing VBL with MnCl_2_ and then oxidized to generate VBL/MnO_2_ under alkaline pH conditions because NaOH can trigger the following oxidation reaction (2MnCl_2_ + 4NaOH + O_2_ → MnO_2_ + 4NaCl + 4H_2_O) [22]. During this process, the hydrophobic units of VBL would be further adsorbed onto the surface of the synthesized VBL/MnO_2_, ultimately yielding VBL/MnO_2_ nanodrugs.

The particle size distribution, morphologies, and zeta potential of VBL/MnO_2_ nanodrugs were characterized with DLS, TEM, and Zetasizer, respectively (Fig. 1B and S1). The hydrodynamic mean size of VBL/MnO_2_ nanodrugs was 153.1 ± 36.1 nm, and their Z-average size was 141.2 nm. They had a midrange PDI value of 0.228, indicating a relative narrow size distribution. The TEM image revealed that although the synthesized VBL/MnO_2_ nanodrugs were dispersed, they formed larger agglomerates comprising small, irregular particles with diameters of 15–30 nm (Refer to the Inset TEM image in Fig. 1B and S2). Moreover, the sizes of VBL/MnO_2_ agglomerates were similar to their hydrodynamic mean sizes determined by DLS. Furthermore, the zeta potential values of free VBL and VBL/MnO_2_ nanodrugs were measured as 0.2 ± 0.1 mV and −12.4 ± 0.7 mV, respectively (Fig. S1). Free VBL exhibited a near-neutral surface charge, likely because of its salt form, whereas the VBL/MnO_2_ nanodrugs a distinctly negative surface charge, which can be attributed to the intrinsically low point of zero charge of MnO_2_ (below pH 3) [43]. This negative surface charge depicts improved colloidal stability of the VBL/MnO_2_ nanodrugs in aqueous environment.

The synthesis of VBL/MnO_2_ nanodrugs was validated with UV/Vis and ICP-OES analyses. Both VBL and VBL/MnO_2_ similarly displayed characteristic absorption bands at approximately 216 and 274 nm (Fig. 1C) [44]. Owing to surface plasmon resonance, the VBL/MnO_2_ nanodrug solution had a yellowish color and broad absorption peak at 280–350 nm [45,46]. Quantitative analysis utilizing UV/Vis spectrophotometer revealed that 1 mg of VBL/MnO_2_ nanodrugs contained approximately 0.39 mg of VBL. Furthermore, ICP-OES analysis verified that 1 mg of VBL/MnO_2_ nanodrugs contained approximately 0.1178 mg of Mn^2+^ content. These data highlight the successful synthesis of VBL/MnO_2_ nanodrugs and the presence of VBL and Mn contents within the nanodrugs. In addition, quantitative analysis demonstrated that the VBL loading content (wt%) of VBL/MnO_2_ nanodrugs reached 67.76 %, substantially surpassing the typical loading efficiencies of conventional drug delivery systems, which typically exhibit drug loadings below 10 % [38]. Remarkably, this loading capacity is comparable to that of hollow nanostructures (∼69 %), which are widely considered among the most effective platforms for high drug-loading nanomedicines [38]. These results highlight the promising potential of VBL/MnO_2_ nanodrugs as a high drug-loading nanomedicine platform, especially given their facile and scalable synthesis method.

The synthesis of VBL/MnO_2_ nanodrugs was further demonstrated via FT-IR and XPS analyses. In the FT-IR spectra (Fig. 1D), VBL exhibited stretching vibrational peaks at 3440 cm^−1^ (O-H) and 1750 cm^−1^ (C=O). In contrast, VBL/MnO_2_ nanodrugs induced the reduction of these O-H and C=O stretching vibrations because of the molecular interactions between the drug and MnO_2_, including the presence of the drug within the VBL/MnO_2_ nanostructures [47]. Moreover, they displayed the characteristic peaks at 492 cm^−1^ owing to Mn-O stretching vibrations in MnO_2_ [48]. In XPS spectra (Fig. 1E), the elemental compositions of C, N, and O atoms were acquired in VBL. Meanwhile, in addition to the elemental compositions of C and O atoms, VBL/MnO_2_ exhibited two distinctive Mn2p peaks at 642.2 eV (Mn 2P3/2) and at 654 eV (Mn2p1/2), corresponding to the binding energies of MnO_2_ [26,49,50]. Further, the difference in binding energies (11.8 eV) between two distinctive Mn2p peaks indicated that the chemical state of the Mn element is Mn^4+^ in the form of MnO_2_ [51]. These results generally depict the successful synthesis of VBL/MnO_2_ nanodrugs and the presence of VBL within them.

### 3.2 Particle stability of VBL/MnO_2_ nanodrugs

The stability of VBL/MnO_2_ nanodrugs was initially evaluated by examining the precipitation and changes in Z-average sizes under various solutions. Aligning with our previous studies [26,50], VBL/MnO_2_ nanodrugs in 0.9 % NS and PBS solutions were quickly aggregated to form larger precipitates (Fig. S2). A marked increase was also detected in their Z-average sizes during the storage under 0.9 % NS and PBS solutions (Fig. 1F), which was attributed to the alkaline cations-induced adsorption of phosphates to the MnO_2_ between pH 6 and 9 [52] or the salt-induced aggregation of MnO_2_ [26,52]. However, no visible precipitates of the VBL/MnO_2_ nanodrugs were detected in DW and 10 % FBS solutions for 96 h (Fig. S3). Furthermore, the Z-average sizes of VBL/MnO_2_ nanodrugs remained constant during the storage in DW and 10 % FBS at 25 °C (Fig. 1F). Notably, storage of the nanodrugs in DW at 4 °C for 30 days did not lead to any significant size change in their Z-average sizes (Fig. S4). These results indicate that the VBL/MnO_2_ nanodrugs remain stable in DW and 10 % FBS solutions during storage.

### 3.3 *In vitro* degradation and oxygen generation of VBL/MnO_2_ nanodrugs under TME-mimic conditions

Endogenous stimuli in the TME, such as acidic pH, high H_2_O_2_, and high GSH, have been utilized to design smart, multifunctional nanomaterials and achieve specifically targeted delivery and controlled drug release in the TME of solid tumors [10,53]. As previously reported, MnO_2_ nanomaterials remain stable under neutral and basic pH, but undergo rapid degradation in the presence of endogenous stimuli [[25], [26], [27], [28]]. We specifically engineered multifunctional and endogenous stimuli-responsive VBL/MnO_2_ nanodrugs to enhance therapeutic responses to hypoxic tumors. Accordingly, the *in vitro* degradation of VBL/MnO_2_ nanodrugs under endogenous H_2_O_2_ level (200 μM) and intracellular GSH level (2 mM) in tumors at pH 7.4 (normal tissue) or acidic pH 6.0 conditions was first investigated by monitoring the absorbance at 320 nm, the maximum absorption wavelength of MnO_2_ (Fig. 1G) [45]. The absorbances of VBL/MnO_2_ nanodrugs at pH 7.4 and 6.0 decreased slowly, and their absorbances at pH 7.4 in the presence of 200 μM H_2_O_2_ or 2 mM GSH decreased marginally within 10 min compared to those at pH 7.4 or 6.0 conditions. However, their absorbances at pH 6.0 with 200 μM H_2_O_2_ decreased rapidly compared to those at pH 7.4 with 200 μM H_2_O_2_. More importantly, the absorbance of VBL/MnO_2_ nanodrugs decreased remarkably faster and more significantly at pH 6.0 with a higher intracellular GSH level than at pH 7.4 with a similar intracellular GSH level. These findings indicate that endogenous stimuli, such as acidic pH, GSH, and H_2_O_2_, rapidly accelerate the degradation of VBL/MnO_2_ nanodrugs in the TME compared to normal physiological conditions.

MnO_2_ nanomaterials can decompose endogenous H_2_O_2_ to generate O_2_, alleviating tumor hypoxia [22,23]. As demonstrated in Fig. 1G, the VBL/MnO_2_ nanodrugs are H_2_O_2_-responsive nanoplatforms under acidic conditions. Therefore, the VBL/MnO_2_ nanodrugs were reacted with different H_2_O_2_ concentrations in N_2_-purged PBS under hypoxia-mimicking conditions to further confirm the possibility of O_2_ generation. Owing to H_2_O_2_ responsiveness of MnO_2_ nanomaterials [22,23], the proposed H_2_O_2_-responsive VBL/MnO_2_ nanodrugs could enhance the *in situ* oxygen generation efficiency in a H_2_O_2_ concentration-dependent manner under hypoxic conditions (Fig. 1H). This implies that the high oxygen-generating capacity of VBL/MnO_2_ nanodrugs can alleviate the hypoxic microenvironment.

### 3.4 *In vitro* intracellular H_2_O_2_ levels in SCC7 cells under normoxic and hypoxic conditions

**Fig. 2:**
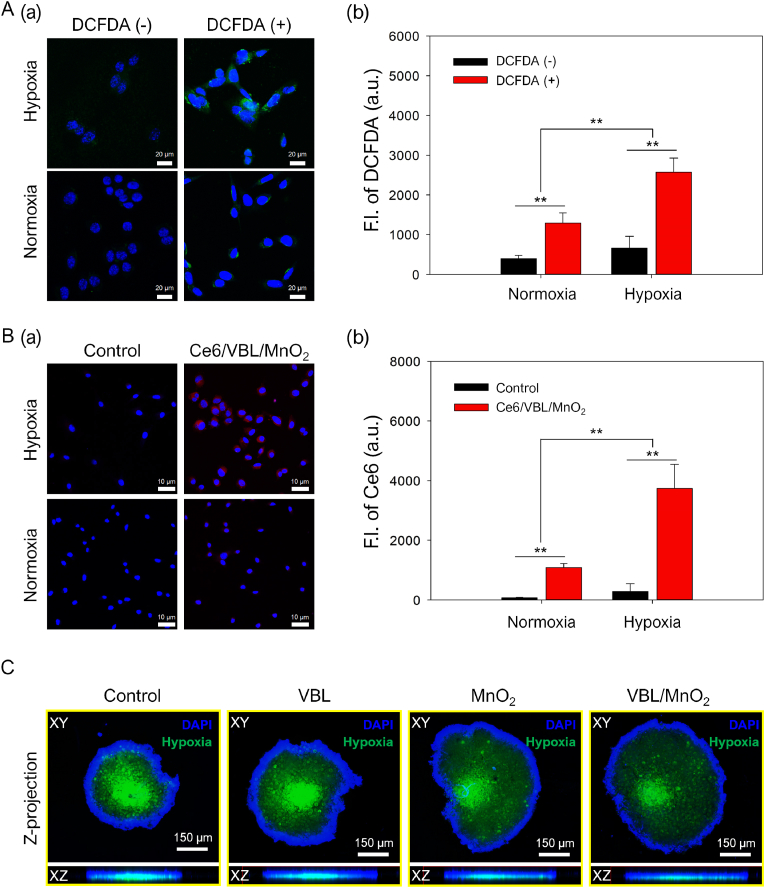
In vitro intracellular uptake and hypoxia alleviation of VBL/MnO_2_ nanodrugs in normoxic and hypoxic SCC7 cells. **(**A) Intracellular H_2_O_2_ detection in normoxic and hypoxic SCC7 cells. (a) Confocal images and (b) quantitative analysis of intracellular H_2_O_2_ levels in normoxic and hypoxic SCC7 cells. Scale bar: 20 μm, ∗∗P < 0.01. (B) Intracellular uptake of Ce6/VBL/MnO_2_ in normoxic and hypoxic SCC7 cells. (a) Confocal images and (b) quantitative analysis for intracellular uptake of Ce6/VBL/MnO_2_ in normoxic and hypoxic SCC7 cells. Scale bar: 10 μm, ∗∗P < 0.01. (C) Comparison of hypoxia alleviation by VBL, MnO_2_, and VBL/MnO_2_ nanodrugs in hypoxic SCC7 tumor spheroids. Confocal Z-stack projection images of SCC7 tumor spheroids following treatment with VBL (5 μM), MnO_2_ (6.1 μM), or VBL/MnO_2_ nanodrugs (5 μM VBL and 6.1 μM MnO_2_). Orthogonal XY and XZ Z-projection images were generated by merging serial scans of 2.5 μm-thick optical sections. Scale bar: 150 μm.

Tumor cells produce high levels of reactive oxygen species (ROS) (specifically H_2_O_2_) than normal cells [54], and hypoxic tumor cells produce higher levels of ROS than normoxic cells [27,55]. Therefore, we investigated and compared the intracellular H_2_O_2_ levels of SCC7 cells under normoxic and hypoxic conditions utilizing the DCFDA dye. The fluorescence-based DCFDA is prevalent for detecting intracellular ROS (i.e., H_2_O_2_, hydroxyl radicals, and peroxynitrite) because DCFDA is converted into weak-fluorescent 2′,7′-dichlorodihydrofluorescein in normoxic cells; however, it is transformed into the highly fluorescent 2′,7′-dichlorofluorescein in hypoxic cells under oxidation by ROS [56]. As illustrated in Fig. 2A(a), the normoxic SCC7 cells with DCFDA exhibited increased fluorescence intensity than the normoxic SCC7 cells without DCFDA. Notably, the hypoxic SCC7 cells treated with DCFDA exhibited a significantly higher fluorescence signal (approximately 1.99-fold) than the normoxic SCC7 cells treated with DCFDA (Fig. 2A(a) and 2A(b)). Consistent with our previous study [27], the data also strongly support the notion that hypoxic tumor cells possess higher intracellular ROS levels than normoxic tumor cells.

### 3.5 *In vitro* intracellular uptake and hypoxia-alleviation effects of VBL/MnO_2_ nanodrugs under normoxic and hypoxic conditions

To monitor the intracellular uptake of VBL/MnO_2_ nanodrugs, we prepared near-infrared fluorescent (NIRF)-emitting Ce6/VBL/MnO_2_ nanodrugs by adsorbing hydrophobic Ce6, a NIRF dye, onto the VBL/MnO_2_ nanodrugs (Fig. S5A). The resulting Ce6/VBL/MnO_2_ nanodrugs contained 0.15 mg Ce6 per milligram of Ce6/VBL/MnO_2_ nanodrugs. However, Ce6 retained its characteristic peaks, including a strong Soret band at 405 nm and Q-bands at 480–700 nm (Fig. S5B). Notably, the UV/Vis spectra in Fig. S5B indicate that the broad absorption of MnO_2_ materials (with the strongest absorption peak at 370 nm) in Ce6/VBL/MnO_2_ nanodrugs, ranging from 250 to 500 nm, overlaps with the strong Soret band of Ce6 [57,58]. In addition, Ce6/VBL/MnO_2_ nanodrugs exhibited a bathochromic shift (approximately 10 nm) in the Q-band absorption peaks (Fig. S5B), attributed to the microenvironmental changes during the formation of Ce6/VBL/MnO_2_ [59]. More importantly, a substantially weak NIRF fluorescence emission of Ce6/VBL/MnO_2_ nanodrugs at 670 nm was observed in the absence of H_2_O_2_ (Fig. S5C) owing to the fluorescence quenching of Ce6 by the proximal distance of between the adsorbed Ce6 molecules on the nanodrugs. However, upon exposure to H_2_O_2_, a significant NIRF signal recovery was observed resulting from the dequenching mechanism obtained via the release of Ce6 after MnO_2_ degradation by H_2_O_2_ (Fig. S5A and S5C) [50,60]. These findings confirm that the successfully prepared H_2_O_2_-responsive Ce6/VBL/MnO_2_ nanodrugs are beneficial in tracking the intracellular uptake of the nanodrugs in tumor cells.

To further validate the hypoxia-alleviation effects of VBL/MnO_2_ nanodrugs in hypoxic tumor cells, the intracellular uptake of Ce6/VBL/MnO_2_ nanodrugs was examined via confocal imaging. Confocal imaging revealed markedly brighter fluorescence intensity of the Ce6/VBL/MnO_2_ group in the cytoplasm of hypoxic SCC7 cells than in normoxic SCC7 cells (Fig. 2B(a)), indicating the release of Ce6 upon degradation of the nanodrugs in hypoxic tumor cells. Quantitative analysis consistently supported these findings, confirming that they exhibited higher fluorescence intensity in hypoxic SCC7 cells than in normoxic SCC7 cells (Fig. 2B(b)). These results indicated that more drug release occurred via the rapid degradation of Ce6/VBL/MnO_2_ nanodrugs following their intracellular uptake into hypoxic tumor cells with higher intracellular H_2_O_2_ levels.

Given the endogenous stimuli-responsiveness of VBL/MnO_2_ nanodrugs, characterized by high oxygen-generating capacity and rapid degradation in hypoxic tumor cells, the *in vitro* hypoxia alleviation effect of VBL/MnO_2_ nanodrugs was investigated utilizing a hypoxia reagent as a fluorogenic compound that becomes fluorescent in hypoxic environments and hypoxic tumor cells [27,40]. As illustrated in the confocal imaging of Fig. S6A, weak or negligible FIs were observed in the normoxic SCC7 of all treated groups (Fig. S6A and S6B). In contrast, strong fluorescence signals were detected in non-treated and VBL-treated hypoxic SCC7 cells (Fig. S6A and S6C), confirming the absence of hypoxia alleviation effect of VBL. However, confocal imaging and quantitative fluorescence analysis revealed a significant reduction of up to approximately 31 and 35 % in fluorescence intensity in VBL-treated hypoxic SCC7 cells after treatment with MnO_2_ and VBL/MnO_2_ nanodrugs (Fig. S6A and S6C). These findings indicate their excellent hypoxia alleviation effect, which is attributable to effective oxygen generation by MnO_2_ of VBL/MnO_2_ nanodrugs following their degradation in hypoxic tumor cells with low oxygen and high H_2_O_2_ contents.

To further substantiate the hypoxia-alleviating effect of VBL/MnO_2_ observed in 2D SCC7 cell cultures, we conducted additional experiments utilizing a 3D tumor spheroid model under hypoxic conditions. As illustrated in Fig. 2C, strong and distinct fluorescence signals, indicative of substantial hypoxic regions, were prominently observed in both the control and free VBL-treated spheroids. A clear gradient of increasing fluorescence intensity from the spheroid periphery toward the core underscored the severity of hypoxia at the spheroid center. In contrast, spheroids treated with MnO_2_ or VBL/MnO_2_ nanodrugs exhibited substantially reduced fluorescence intensity, signifying effective hypoxia alleviation within the spheroid core. These findings reinforce the results from the 2D cell model and highlight the potential of MnO_2_-containing nanodrugs to generate oxygen within the TME, thereby mitigating hypoxia and potentially enhancing therapeutic efficacy in hypoxic tumors.

### 3.6 *In vitro* antitumor effects of VBL/MnO_2_ nanodrugs against normoxic and hypoxic tumor cells

**Fig. 3:**
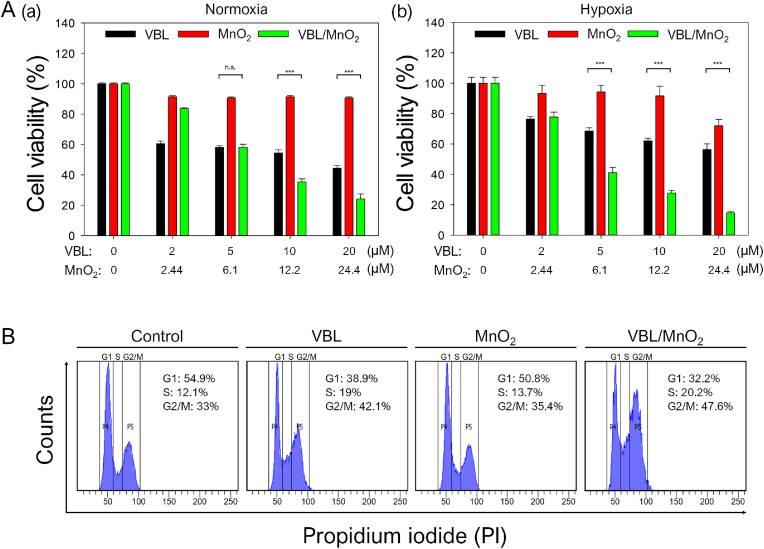
*In vitro* antitumor effects. (A) *In vitro* therapeutic effects of VBL, MnO_2_, and VBL/MnO_2_ nanodrugs on (a) normoxic and (b) hypoxic SCC7 cells. ∗∗∗P < 0.001. (B) Flow cytometry histograms illustrating the cell cycle distribution of hypoxic SCC7 cells, assessed by propidium iodide (PI)-based flow cytometry after treatment with VBL (5 μM), MnO_2_ (6.1 μM), or VBL/MnO_2_ nanodrugs (equivalent to 5 μM VBL and 6.1 μM MnO_2_).

Hypoxic TMEs, which are prevalent in most solid tumors, are well known to diminish tumor cell sensitivity [13] and promote therapeutic resistance to chemotherapy [7]. To determine whether hypoxia relief by VBL/MnO_2_ nanodrugs improves antitumor efficacy, we compared the cytotoxic effects of VBL, MnO_2_, and VBL/MnO_2_ nanodrugs on normoxic and hypoxic SCC7 cells. MnO_2_ alone exhibited negligible toxicity under both conditions up to 12.2 μM concentration (Fig. 3A). As anticipated, free VBL reduced the viability of normoxic SCC7 cells in a dose-dependent manner (Fig. 3A(a)). However, hypoxic SCC7 cells displayed greater viability upon VBL treatment compared to normoxic cells (Fig. 3A(b)), likely because of the reduced VBL responses against hypoxic tumor cells compared to normoxic tumor cells [13]. Remarkably, VBL/MnO_2_ nanodrugs exhibited higher therapeutic responses at VBL concentrations above 10 μM under normoxia and above 5 μM under hypoxia (Fig. 3A(a) and 3A(b)). This improved efficacy is attributed to the hypoxia-responsive degradation of MnO_2_ within tumor cells, where elevated intracellular H_2_O_2_ levels promote oxygen generation that alleviates hypoxia and triggers rapid release of VBL from the nanodrugs [23]. Complementary flow cytometry analysis revealed that VBL/MnO_2_ nanodrugs induced a greater G_2_/M cell cycle arrest in hypoxic SCC7 cells compared to free VBL (Fig. 3B), indicating enhanced mitotic disruption and cell death due to hypoxia alleviation and controlled drug release. Overall, these findings demonstrate that VBL/MnO_2_ nanodrugs enhance therapeutic responses in hypoxic tumor cells more effectively than free VBL, by combining hypoxia relief with efficient VBL delivery.

### 3.7 *In vitro* microtubule depolymerization of VBL/MnO_2_ nanodrugs against normoxic and hypoxic tumor cells

Microtubules constitute the primary dynamic structural components of cell's cytoskeleton and represent key targets for various anticancer drugs [61]. However, hypoxia alone can induce microtubule depolymerization within tumor cells [62]. This disruption of microtubule networks under hypoxic conditions may compromise cell viability and reduce the effectiveness of microtubule-targeting agents by lowering the abundance of intact microtubules available for drug binding. Consistent with the previous report, hypoxic SCC7 cells exhibited a time-dependent loss of microtubules adjacent to the plasma membrane compared to the well-organized microtubule networks observed in normoxic SCC7 cells (Fig. S7). After 1 h of hypoxia, microtubule loss was detected. By 4 h, a significant reduction in polymerized microtubules was observed, with only fragmented structures remaining. After 24 h, microtubule structures were nearly absent. These findings suggest that hypoxia induced microtubule depolymerization and loss of microtubules in tumor cells.

**Fig. 4:**
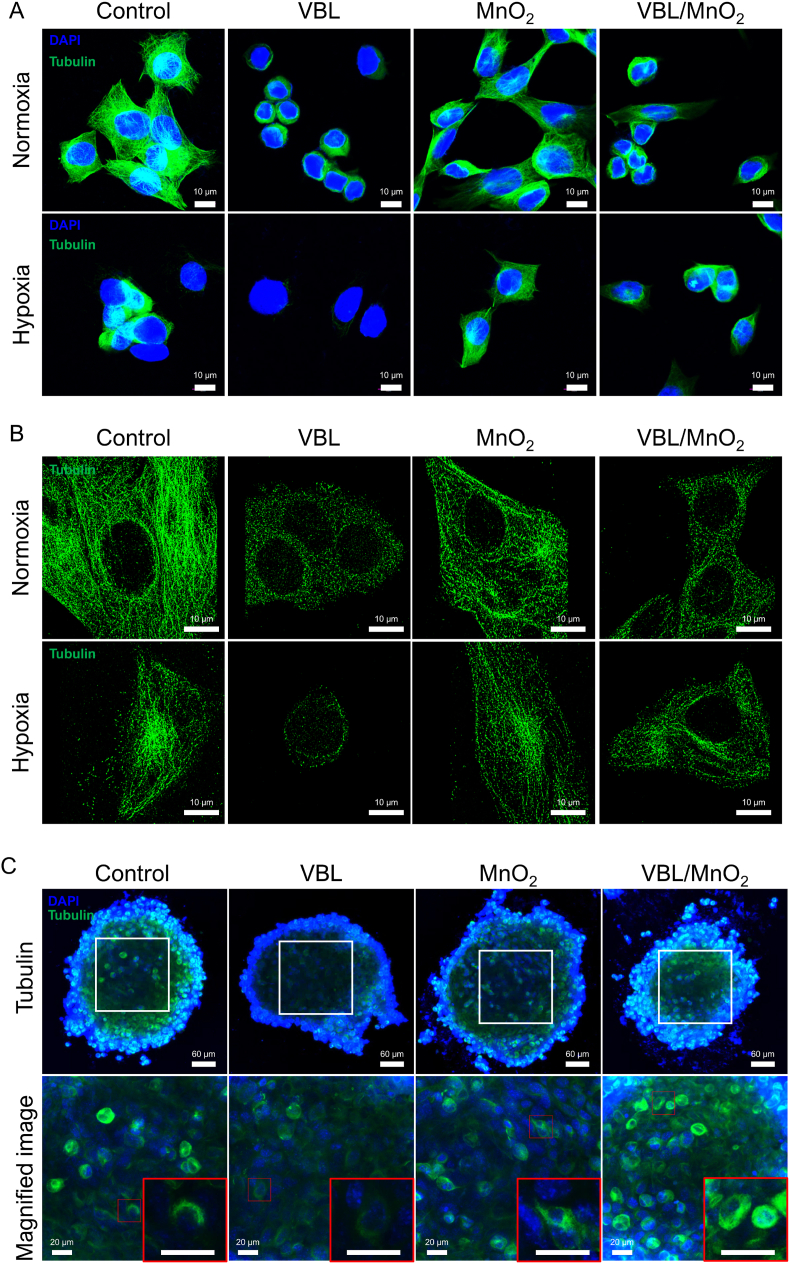
*In vitro* microtubule depolymerization of VBL/MnO_2_ nanodrugs in normoxic and hypoxic SCC7 cells. (A) Fluorescence images and (B) STORM (stochastic optical reconstruction microscopy) images illustrating the microtubule depolymerization in SCC7 cells under normoxic and hypoxic conditions following treatment with VBL (5 μM), MnO_2_ (6.1 μM), or VBL/MnO_2_ nanodrugs (equivalent to 5 μM VBL and 6.1 μM MnO_2_). Scale bar: 10 μm. (C) Representative CLSM images evaluating the microtubule depolymerization effects of VBL/MnO_2_ nanodrugs in a 3D SCC7 tumor spheroid model under hypoxic conditions following treatment with VBL (5 μM), MnO_2_ (6.1 μM), or VBL/MnO_2_ nanodrugs (equivalent to 5 μM VBL and 6.1 μM MnO_2_). Z-stack projection images were generated by merging serial scans of 2.5 μm-thick optical sections. Scale bar: 60 μm. Insets display magnified views with scale bar of 20 μm.

VBL, a microtubule inhibitor, disrupts microtubule assembly in tumor cells, causing microtubule depolymerization [8,9,11,12]. To examine detailed alterations in the microtubule network in normoxic and hypoxic SCC7 cells with or without drug treatment, we employed both CLSM and STORM, a single-molecule super-resolution technique offering approximately tenfold higher resolution than conventional fluorescence microscopy [63]. As illustrated in Fig. 4A and B, microtubules in control normoxic SCC7 cells exhibited a well-organized, linear filamentous structure. Similarly, normoxic SCC7 cells treated with MnO_2_, which lacks microtubule-disrupting properties, maintained a microtubule network closely resembling that of the control group. In contrast, treatment with either VBL or VBL/MnO_2_ nanodrugs demonstrated pronounced disruption of the microtubule network, evidenced by fragmented polymerized tubulin. Consistent with CLSM images in Fig. S7 and previous reports [62], control hypoxic SCC7 cells exhibited less regular microtubule structures, reduced microtubule density, and a loss of microtubules near the plasma membrane. Therefore, VBL-treated hypoxic SCC7 cells exhibited further microtubule reduction relative to both control hypoxic cells and VBL-treated normoxic cells, likely due to an already compromised microtubule network under hypoxia. Meanwhile, MnO_2_ treatment in hypoxic SCC7 cells significantly restored the microtubule network by alleviating hypoxia, as indicated by the reappearance of orderly, straight microtubule segments. Importantly, treatment with VBL/MnO_2_ nanodrugs in hypoxic SCC7 cells significantly disrupted this restored microtubule network (Fig. 4A and B), which may be attributed both to hypoxia alleviation by MnO_2_ and the release of the microtubule-disrupting vinblastine from the nanodrugs.

Furthermore, both VBL and VBL/MnO_2_ nanodrug treatments induced significant nuclear condensation (Fig. 4A). As previously reported [64], microtubule-disrupting agents such as VBL trigger nuclear condensation in tumor cells, a morphological change typically recognized as a reduction in nuclear size. Consistent with this, Fig. 4A demonstrates that under normoxic conditions, SCC7 cells treated with VBL or VBL/MnO_2_ nanodrugs exhibited reduced nuclear size, likely reflecting their microtubule-disrupting activity. Notably, VBL alone did not induce nuclear condensation under hypoxic conditions. However, SCC7 cells treated with VBL/MnO_2_ nanodrugs under hypoxia exhibited significant nuclear condensation, which may be attributed to the improved antitumor efficacy of VBL/MnO_2_ in hypoxic SCC7 cells.

Subsequently, the microtubule depolymerization effects of VBL/MnO_2_ nanodrugs were further evaluated utilizing a 3D SCC7 tumor spheroid model under hypoxic conditions to better mimic the TME. As demonstrated in Fig. 4C, control spheroids exhibited relatively strong fluorescence signal at the spheroid periphery, indicating the presence of microtubule structures. However, a continuous decrease in fluorescence intensity toward the spheroid core was observed, reflecting hypoxia-induced microtubule depolymerization and a loss of microtubules in the central region, consistent with those observed in Figs. S7, 4A, and 4B. Treatment with VBL alone, resulted in significant decrease in fluorescence intensity throughout the spheroids, indicating enhanced microtubule depolymerization due to both hypoxia and VBL's targeted action on microtubules, particularly at the spheroid periphery. Importantly, the hypoxic core, characterized by pre-existing microtubule disassembly or destabilization, demonstrated a limited response to VBL treatment, indicating reduced drug efficacy in these regions and highlighting the inherent limitation of microtubule-targeting agents within hypoxic TME. However, spheroids treated with MnO_2_ alone or VBL/MnO_2_ nanodrugs exhibited partial restoration of microtubule structures at both the spheroid periphery and core. This effect is attributed to MnO_2_-mediated oxygen generation, which alleviates hypoxia and facilitates the reassembly of depolymerized microtubules. As illustrated in Fig. 4A and B, spheroids treated with VBL/MnO_2_ nanodrug exhibited notably greater microtubule disruption and increased tubulin aggregation compared to those treated with MnO_2_ alone. This enhanced disruption and aggregation likely results from the combined effects of hypoxia alleviation by MnO_2_ and targeted action of released VBL on microtubules within the hypoxic TME. These findings confirm that VBL/MnO_2_ nanodrugs effectively overcome the inherent limitations posed by hypoxia-mediated resistance to microtubule-targeting agents in hypoxic tumor regions by restoring drug targets via oxygen generation and delivering vinblastine for improved cytotoxic efficacy.

### 3.8 *In vivo* tumor accumulation and hypoxia alleviation effects of nanodrugs on SCC7-tumor-bearing mice

**Fig. 5:**
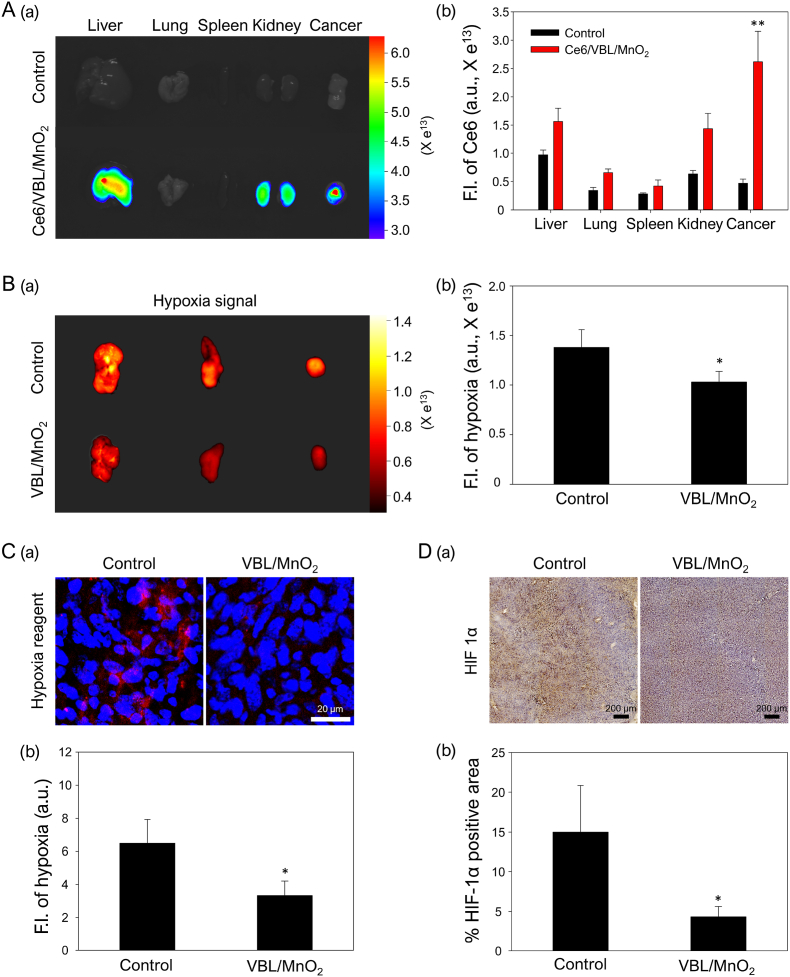
*In vivo* tumor accumulation and amelioration of hypoxia. (A) Tumor accumulation of Ce6/VBL/MnO_2_ nanodrugs into SCC7-tumor-bearing mice. (a) *Ex vivo* fluorescence images and (b) quantitative FIs of excised tumors and major organs (i.e., liver, lung, spleen, and kidney) 24 h after i.v. injection of PBS or Ce6/VBL/MnO_2_ nanodrugs. ∗∗P < 0.01. (B) Hypoxia alleviation effects of VBL/MnO_2_ nanodrugs on SCC7 tumor-bearing mice. (a) *Ex vivo* hypoxia imaging and (b) quantitative analysis of isolated tumors from mice. ∗P < 0.05. (C) (a) Confocal images and (b) quantitative analysis for hypoxia signals of sectioned tumors in control and VBL/MnO_2_ groups. Scale bar: 20 μm, ∗P < 0.05. (D) (a) Immunohistochemistry of HIF-1α and (b) quantitative analysis of HIF-1α positive area (%) in tumor slices of control and VBL/MnO_2_ group. Scale bar: 200 μm, ∗P < 0.05.

To investigate *in vivo* tumor accumulation, PBS or Ce6/VBL/MnO_2_ nanodrugs (2 mg/kg of Ce6) were administered intravenously to SCC7-tumor-bearing mice, and *ex vivo* fluorescence imaging of excised tumors and major organs (i.e., liver, lung, spleen, and kidney) was conducted at 24 h post-injection. No fluorescence signal was observed in any of the tissues of the PBS-treated control group, whereas fluorescence signals were detected in the liver, kidney, and tumors of the Ce6/VBL/MnO_2_-treated group (Fig. 5A(a) and 5A(b)). In particular, the fluorescence intensity of tumor tissues was stronger than that of liver and kidney tissues. Furthermore, *ex vivo* quantitative fluorescence analysis revealed that the fluorescence signals of tumor tissues in the Ce6/VBL/MnO_2_-treated group were approximately 5.6 times higher than those in the PBS-treated control group (Fig. 5A(b)). Moreover, the confocal images and quantitative analysis of the tumor sections revealed consistent results, with a more intense fluorescence in the Ce6/VBL/MnO_2_-treated group than in the control group (Fig. S8A and S8B), highlighting the substantial accumulation of Ce6/VBL/MnO_2_ nanodrugs in SCC7 tumor tissues. These results indicate that the nanodrugs possess *in vivo* tumor-homing properties via passive tumor targeting due to the enhanced permeation and retention effect in tumor tissues [65,66].

Considering the oxygen-generating ability of VBL/MnO_2_ nanodrugs, which possess tumor-homing properties, we further confirm the *in vivo* hypoxia-alleviation ability of VBL/MnO_2_ nanodrugs in SCC7-tumor-bearing mice utilizing a hypoxia reagent (as a sensitive oxygen detector) and an HIF-1α antibody (as a classic hypoxia marker). As illustrated in *ex vivo* fluorescence images and quantitative analysis (Fig. 5B(a) and 5B(b)), the strong tumor hypoxia signal in the control group was significantly decreased by approximately 25 % after treatment with VBL/MnO_2_ nanodrugs. Compared to the control group, the tumor slices from the VBL/MnO_2_-treated group exhibited a significant reduction in red hypoxia fluorescence, reaching approximately 48.8 % (Fig. 5C(a) and 5C(b)). These results suggest that oxygen generation via the degradation of VBL/MnO_2_ nanodrugs accumulated in the tumor can effectively reduce tumor hypoxia. Meanwhile, HIF-1α is recognized as a key mediator of hypoxia signaling, and the increased HIF-1α expression is associated with hypoxia-induced chemoresistance in cancer therapy [7,67]. As illustrated in immunohistochemistry analysis (Fig. 5D(a)), high HIF-1α expression was observed in the control group, whereas its expression was remarkably decreased in the VBL/MnO_2_-treated group. Furthermore, quantitative analysis confirmed that the VBL/MnO_2_-treated group significantly reduced the HIF-1α positive area up to approximately 3.47-fold compared to the PBS-treated control group (Fig. 5D(b)). The data further support the notion that hypoxia relief by VBL/MnO_2_ nanodrugs can effectively downregulate HIF-1α expression in tumor tissues, as previously reported [49,68].

### 3.9 *In vivo* anticancer effects of VBL/MnO_2_ nanodrugs on SCC7-tumor-bearing mice

**Fig. 6:**
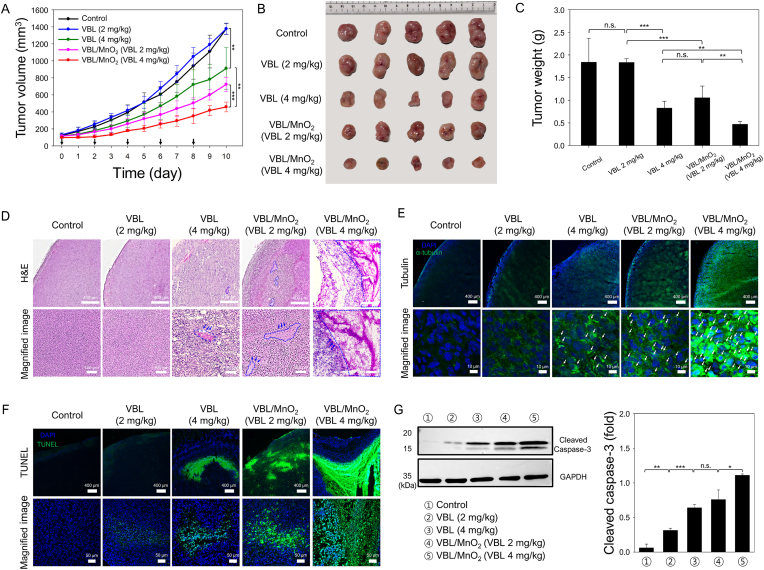
*In vivo* therapeutic effects of VBL/MnO_2_ nanodrugs on SCC7-tumor-bearing mice. (A) Tumor volumes of SCC7-tumor-bearing mice after intravenously receiving PBS (control), VBL (2 and 4 mg/kg), and VBL/MnO_2_ (2 and 4 mg VBL/kg) once every 2 days. Data represent mean ± S.D. (n = 5/group). ∗∗∗P < 0.001. (B) Photographs and (C) weights of excised tumors from mice in each group at the end of the experiment. ∗P < 0.05, ∗∗∗P < 0.001, n.s. = no significance. (D) H&E staining of tumor tissues after treatment. Scale bar: 400 μm. In magnified images, acellular regions are marked with the blue dotted line. Scale bar: 100 μm. (E) Representative immunofluorescence images of tubulin in tumor slices in each group. Scale bar: 400 μm. Tubulin aggregation in magnified images is represented by white arrows. Scale bar: 10 μm. (F) Representative images of TUNEL staining of tumor tissues. Scale bar: 400 μm. Magnified TUNEL images revealed apoptotic-positive tumor cells (green) after treatment. Scale bar: 50 μm. Nuclei are stained blue (DAPI), and apoptotic cells are stained green (TUNEL). (G) Assessment and quantification of apoptosis-associated cleaved caspase-3 levels by western blot analysis. ∗P < 0.05, ∗∗P < 0.01, ∗∗∗P < 0.001, n.s. = no significance. (For interpretation of the references to color in this figure legend, the reader is referred to the Web version of this article.)

The *in vivo* antitumor effects of VBL/MnO_2_ nanodrugs were studied with the SCC7 solid tumor model. When the tumor volumes reached 100−150 mm^3^, PBS, VBL (2 and 4 mg/kg), and VBL/MnO_2_ nanodrugs (2 and 4 mg VBL/kg) were i.v. injected into randomly selected mice in five groups. The tumor volumes were measured daily for 10 days (Fig. 6A), and the excised tumors from mice in all groups were collected and weighed on Day 10 (Fig. 6B and C).

Free VBL at 2 mg/kg had no significant effect on tumor growth rate compared to the control group, whereas a 4 mg/kg dose resulted in an approximately 32 % reduction. In contrast, treatment with VBL/MnO_2_ nanodrugs led to significantly greater tumor growth inhibition, achieving reductions of approximately 47.5 and 65 % at 2 and 4 mg/kg VBL doses, respectively. These trends were corroborated by tumor photographs and weights, which demonstrated substantial reductions in tumor size and weight for free VBL (4 mg/kg) as well as both doses of VBL/MnO_2_ nanodrugs (Fig. 6B and C). Remarkably, the antitumor efficacy of free VBL at 4 mg/kg was comparable to that of VBL/MnO_2_ nanodrugs at 2 mg/kg, while the VBL/MnO_2_ group at 4 mg/kg exhibited the most pronounced tumor suppression (∼73 % reduction in tumor weight). These results highlight the superior antitumor potency of VBL/MnO_2_ nanodrugs relative to free VBL.

Histological analyses via H&E of tumor sections on Day 10 revealed that control and low-dose VBL (2 mg/kg) groups maintained largely viable tumor morphology (Fig. 6D). Tumors treated with higher-dose VBL (4 mg/kg) and VBL/MnO_2_ nanodrugs (2 and 4 mg/kg) exhibited extensive acellular regions and apoptotic cells with nuclear damage. Immunofluorescence staining for tubulin demonstrated modest aggregation in the low-dose VBL group, whereas, pronounced tubulin aggregation was observed in the high-dose VBL and VBL/MnO_2_ groups, with the strongest signals observed in the 4 mg/kg VBL/MnO_2_-treated tumors (Fig. 6E). These *in vivo* findings suggest that alleviation of tumor hypoxia by VBL/MnO_2_ nanodrugs restores microtubule integrity and enhances vinblastine-mediated microtubule disruption and tubulin aggregation, thereby overcoming hypoxia-induced drug resistance. Consistent with histology, TUNEL assays revealed significantly greater apoptotic responses in tumors from the 4 mg/kg VBL-treated and both VBL/MnO_2_-treated groups compared to the 2 mg/kg VBL group (Fig. 6F). VBL/MnO_2_ nanodrug-treated group at 4 mg/kg of VBL exhibited the highest apoptosis cell counts and fluorescence intensity, demonstrating stronger induction of apoptosis than free VBL alone. Furthermore, Western blot analysis of cleaved caspase-3 further corroborated these findings (Fig. 6G). Slight upregulation of cleaved caspase-3 was observed in the 2 mg/kg VBL group compared to the control, while the 4 mg/kg VBL group exhibited greater expression comparable to the 2 mg/kg VBL/MnO_2_ group. Importantly, the highest cleaved caspase-3 levels were detected in the 4 mg/kg VBL/MnO_2_-treated tumors, indicating dose-dependent apoptosis induction enhanced by the nanodrug formulation.

Previous studies have demonstrated that hypoxia in tumor cells inhibits tubulin aggregation formation and promotes resistance to chemotherapy [7,13]. In line with TUNEL previous reports [8,9], VBL induced microtubule depolymerization, resulting in mitotic arrest and apoptotic cell death in normoxia; however, its efficacy is diminished under hypoxia due to impaired drug activity. Importantly, hypoxia relief via oxygen generation *in situ* has been reported to overcome this resistance mechanism [69]. Our *in vitro* and *in vivo* studies confirm that the VBL/MnO_2_ nanodrugs effectively alleviate tumor hypoxia via *in situ* oxygen generation, thereby overcoming hypoxia-induced drug resistance. The combined effects of hypoxia relief and targeted delivery of vinblastine promote enhanced microtubule depolymerization, extensive tubulin aggregation, mitotic arrest, and apoptosis in SCC7 tumors, as validated in Fig. 4, Fig. 5B/C, and 6. The improved apoptosis induction and tumor regression observed with VBL/MnO_2_ nanodrugs are therefore attributed to enhanced drug efficacy facilitated by hypoxia alleviation and restoration of microtubule dynamics.

### 3.10 *In vivo* biosafety study

The *in vivo* safety concerns associated with nanomaterials are essential for biomedical applications. Mice body weights, serum biochemistry, and histology with H&E staining were also conducted on SCC7-tumor-bearing mice to verify the *in vivo* biosafety of VBL/MnO_2_ nanodrugs. Body weight changes in mice are considered an indicator of drug-induced toxicity because a 20 % or more body weight reduction is considered *in vivo* toxicity [70]. Fig. S9 demonstrates that body weights of all mice across all treatment groups remained stable throughout the experiment, and no mice experienced weight loss of 20 % or more.

**Fig. 7:**
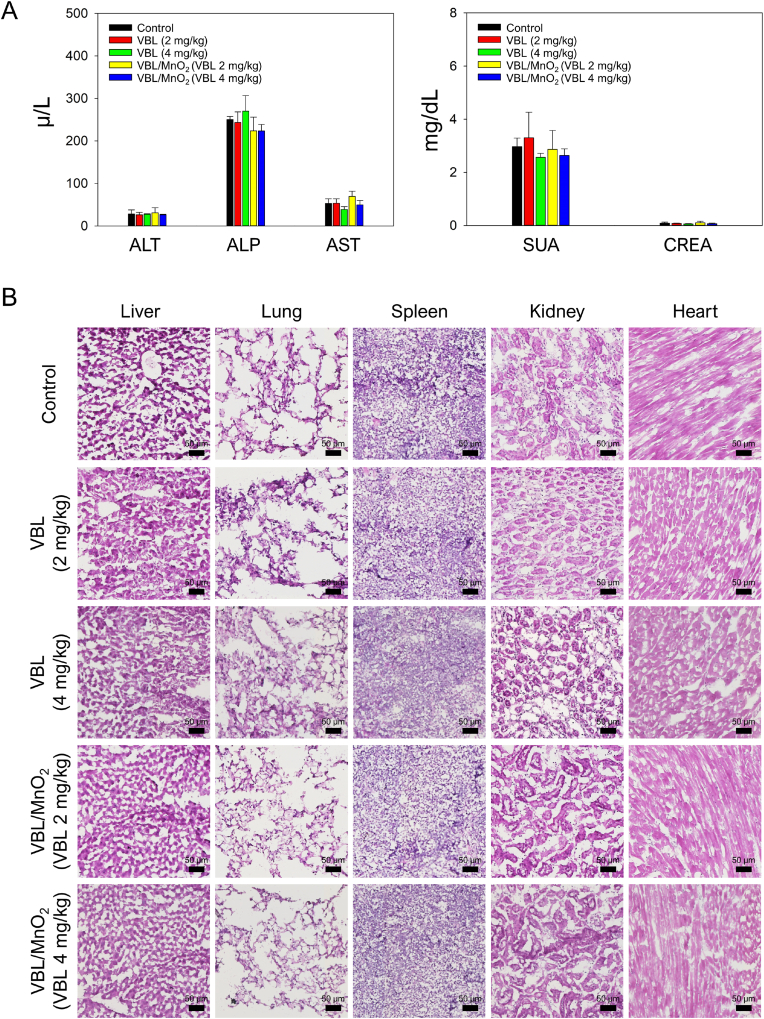
*In vivo* biosafety study. (A) Serum biochemistry analysis of mice after treatment. ALT, ALP, and AST for liver function. SUA and CREA for kidney function. (B) H&E staining of major organs, including liver, lung, spleen, kidney, and heart. Scale bar: 50 μm.

Furthermore, although the accumulation of VBL/MnO_2_ nanodrugs were observed in the liver and kidney (Fig. 5A(a)), the serum biochemistry analysis revealed that the serum biochemical parameters, including ALT, ALP, AST, SUA, and CREA, did not significantly differ in all groups, verifying healthy liver and kidney functions (Fig. 7A). Moreover, H&E images revealed histologically normal tissues in major organs (liver, lung, spleen, kidney, and heart), both in the VBL and VBL/MnO_2_ groups (Fig. 7B). Importantly, the excellent biocompatibility and minimal systemic toxicity of VBL/MnO_2_ nanodrugs may be attributed to the intrinsic biodegradability of MnO_2_-based nanomaterials, which decompose into water-soluble Mn^2+^ ions that are efficiently excreted via the renal pathway [33,34].

## 4 Conclusion

Here, we developed multifunctional and endogenous stimuli-responsive VBL/MnO_2_ nanodrugs for enhanced tumor therapy via a simple reaction of VBL and MnCl_2_ under alkaline pH conditions. The resulting VBL/MnO_2_ nanodrugs exhibited colloidal stability and were readily degraded by endogenous stimuli (i.e., acidic pH, GSH, and H_2_O_2_) in the TME. In addition, H_2_O_2_-responsive VBL/MnO_2_ nanodrugs with high oxygen-generating capacity can effectively alleviate the hypoxic environment, and they also effectively mitigate hypoxia in tumor cells with low O_2_ and high H_2_O_2_ levels after the degradation of internalized VBL/MnO_2_ nanodrugs within hypoxic SCC7 tumor cells. The VBL/MnO_2_ nanodrugs improved therapeutic responses compared to VBL by enhancing drug efficacy and promoting greater microtubule depolymerization and mitotic arrest in hypoxic tumor cells. Furthermore, the accumulation of VBL/MnO_2_ nanodrugs in tumor tissues via passive targeting resulted in the effective reduction of hypoxia and HIF-1α expression *in vivo*. Moreover, the excellent tumor hypoxia relief by biocompatible VBL/MnO_2_ nanodrugs improved drug responses and tubulin aggregation in SCC7 solid tumors, ultimately resulting in enhanced tumor apoptosis and regression. Therefore, these findings underscore the potential of VBL/MnO_2_ nanodrugs as an effective therapeutic strategy for targeting tumor hypoxia and improving therapeutic responses in hypoxic tumors. Notably, this study demonstrated that VBL/MnO_2_ nanodrugs can be synthesized via the interaction of VBL with Mn^2+^ ions and oxidation at an alkaline pH. Given the interaction of various anti-tumor drugs with Mn^2+^, we infer that the drug/MnO_2_ nanoplatforms may become a promising alternative therapeutic strategy for enhancing therapeutic outcomes in hypoxic tumor chemotherapy.

## CRediT authorship contribution statement

**Yong Geun Lim:** Writing – original draft, Methodology, Investigation, Formal analysis, Conceptualization. **Yeji Chang:** Investigation, Formal analysis. **Seon-Ju Park:** Formal analysis, Methodology. **Kyoung-Dong Kim:** Investigation, Writing – review & editing. **Kyeongsoon Park:** Writing – review & editing, Supervision, Funding acquisition, Conceptualization.

## Declaration of competing interest

The authors declare that they have no known competing financial interests or personal relationships that could have appeared to influence the work reported in this paper.

## Data Availability

Data will be made available on request.
