# Supplementary material for: Multifunctional and endogenous stimuli-responsive vinblastine sulfate/manganese dioxide nanodrugs for enhancing chemotherapeutic efficacy against hypoxic tumors
Source: Mater Today Bio. 2025 Aug 23;34:102229. doi: 10.1016/j.mtbio.2025.102229 (PMC12398928; doi:10.1016/j.mtbio.2025.102229)
Supplement: Multimedia component 1 [file mmc1.docx]

***Supplementary Information***

**Multifunctional and endogenous stimuli-responsive vinblastine sulfate/manganese dioxide nanodrugs for enhancing chemotherapeutic efficacy against hypoxic tumors**

Yong Geun Lim^a^, Yeji Chang^a^, Seon-Ju Park^a^, Kyoung-Dong Kim^a^, Kyeongsoon Park^a, *^

^a^ Department of Systems Biotechnology, Chung-Ang University, Anseong, Gyeonggi 17546, Korea

**^*^Corresponding author: Kyeongsoon Park, Ph.D.**

Department of Systems Biotechnology, Chung-Ang University, Anseong, Gyeonggi 17546, Korea; Tel: +82-31-670-3357; Fax: +82-31-675-1381; Email: [kspark1223@cau.ac.kr](mailto:kspark1223@cau.ac.kr)


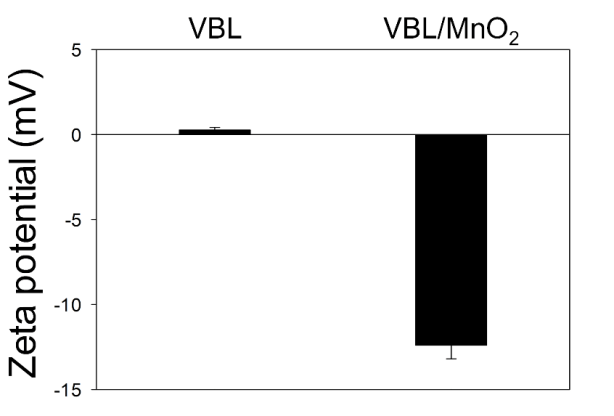


**Fig. S1.** Zeta potential of VBL and VBL/MnO_2_ nanodrugs.


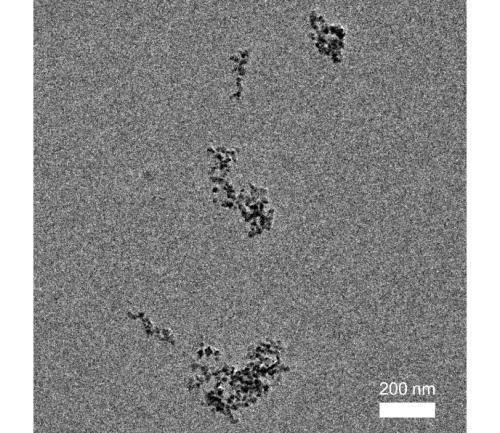


**Fig. S2.** TEM image of VBL/MnO_2_ nanodrugs. Scale bar: 200 nm.


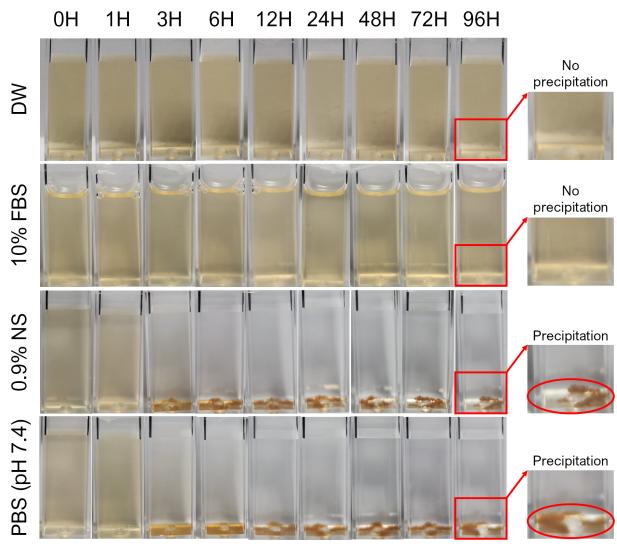


**Fig. S3**. Photos of particle stability of VBL/MnO_2_ nanodrugs under different storage solutions in DW, 10% FBS, 0.9% NS, and PBS (pH 7.4) during 96-h incubation.


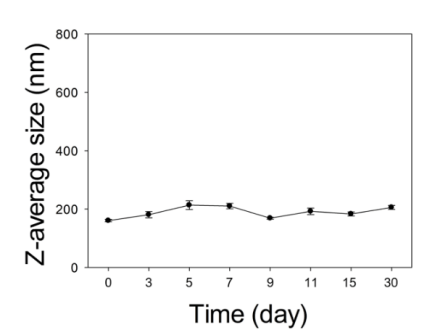


**Fig. S4**. Z-average sizes of VBL/MnO_2_ nanodrugs stored in DW at 4 °C for 30 days.


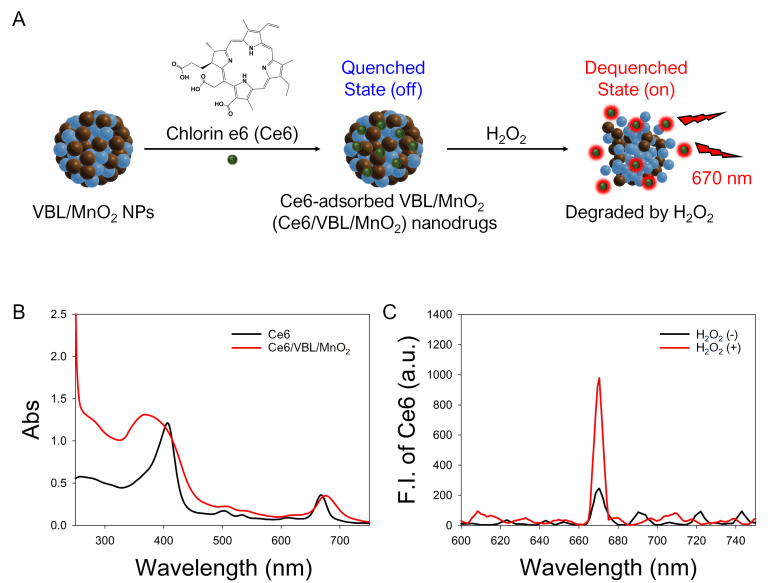


**Fig. S5**. Synthesis and characterizations of Ce6-adsorbed VBL/MnO_2_ (Ce6/VBL/MnO_2_) nanodrugs. (A) Synthesis of Ce6/VBL/MnO_2_ nanodrugs. Hydrophobic and NIRF Ce6 molecules were adsorbed onto the VBL/MnO_2_ nanodrugs, resulting in the formation of Ce6/VBL/MnO_2_ nanodrugs. The Ce6/VBL/MnO_2_ nanodrugs were non-fluorescent in their normal state; however, they emitted strong fluorescence (Em = 670 nm) in the presence of H_2_O_2_. (B) UV/Vis spectra of Ce6 and Ce6/VBL/MnO_2_ nanodrugs. (C) Comparison of fluorescence intensity of Ce6/VBL/MnO_2_ nanodrugs with or without H_2_O_2_.


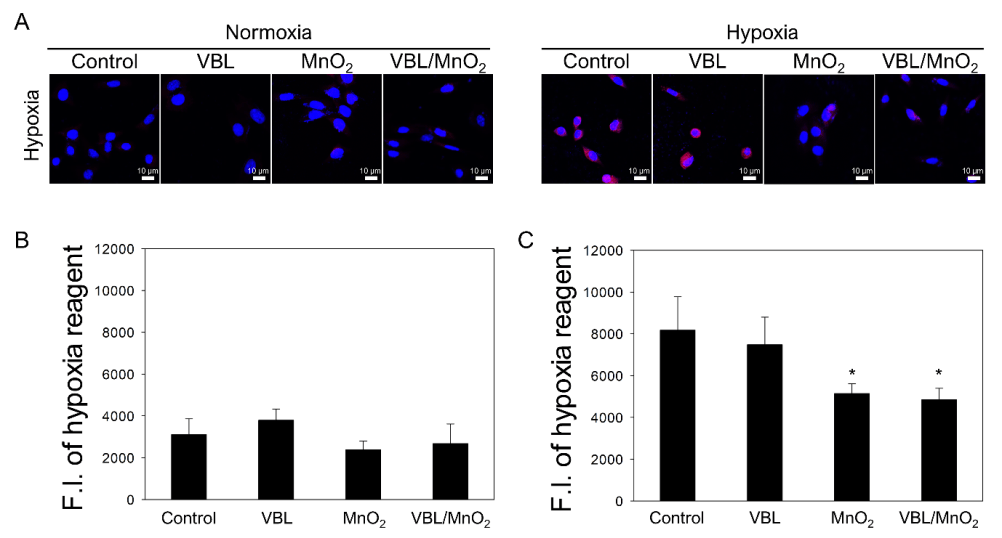


**Fig. S6**. Comparison of hypoxia alleviation by VBL, MnO_2_, and VBL/MnO_2_ nanodrugs in normoxic and hypoxic SCC7 cells. (A) Fluorescence images of normoxic and hypoxic SCC7 cells treated with VBL, MnO_2_, or VBL/MnO_2_ nanodrugs. Scale bar: 10 μm. Quantitative fluorescence signals of (B) normoxic and (C) hypoxic SCC7 cells treated with VBL, MnO_2_, or VBL/MnO_2_ nanodrugs. * P < 0.05.


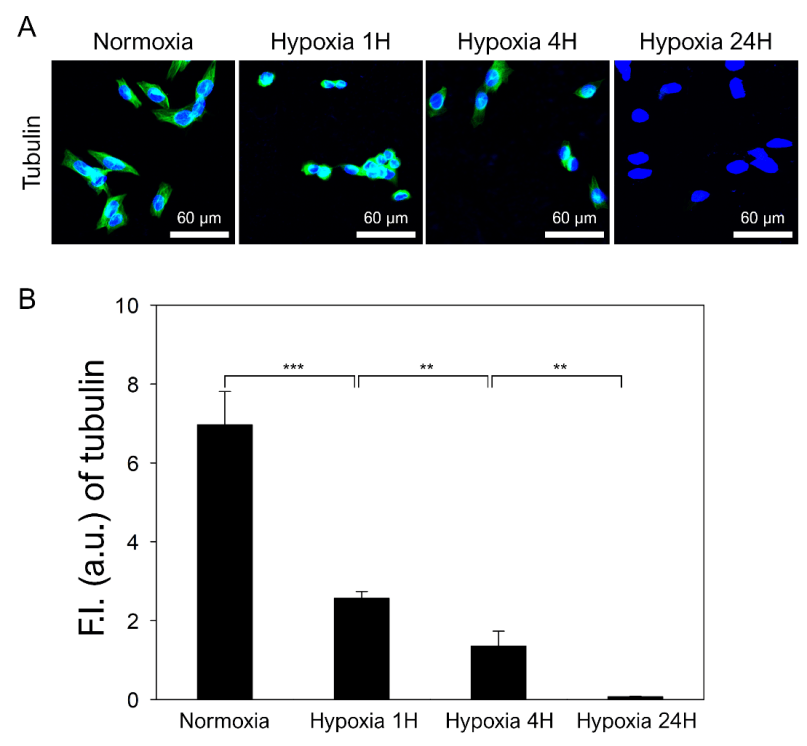


**Fig. S7**. Time-dependent hypoxia-induced microtubule depolymerization in SCC7 cells. (A) Representative fluorescence microscopy images of SCC7 cells cultured under normoxic or hypoxic conditions for 1, 4, and 24 h. representing microtubule structures. Scale bar: Scale bar: 60 μm. (B) Quantitative analysis of fluorescence intensity reflecting microtubule depolymerization under indicated conditions. ** P < 0.01, *** P < 0.001.

**
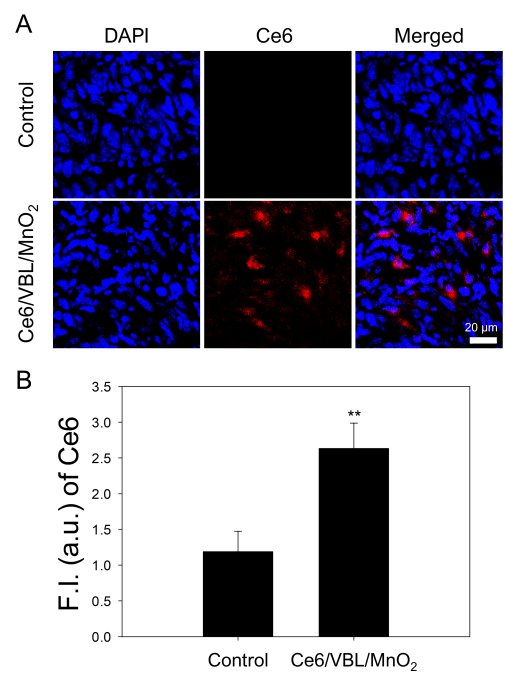
**

**Fig. S8**. Tumor accumulation of Ce6/VBL/MnO_2_ nanodrugs in SCC7 tumor tissues. (A) Confocal images and (B) quantitative fluorescence signals in tumor slices of control and Ce6/VBL/MnO_2_ groups. Scale bar: 20 μm, ** P < 0.01.


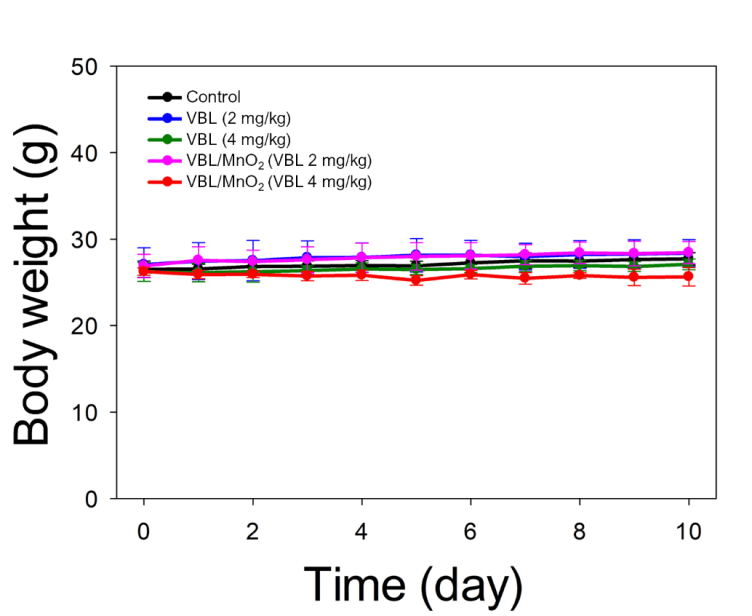


**Fig. S9.** Body weight changes of SCC7 tumor-bearing mice in each group after treatment with PBS (control), free VBL (2 and 4 mg/kg), or VBL/MnO_2_ nanodrugs (equivalent to 2 and 4 mg/kg of VBL). Data represent mean ± S.D. (n =5/group).
